# Introducing antimicrobial stewardship to the outpatient clinics of a suburban academic health system

**DOI:** 10.1017/ash.2021.228

**Published:** 2022-01-17

**Authors:** Travis B. Nielsen, Maressa Santarossa, Beatrice Probst, Laurie Labuszewski, Jenna Lopez, Mary Barsanti-Sekhar, Fritzie Albarillo

**Affiliations:** 1 Loyola University Chicago Stritch School of Medicine, Maywood, Illinois; 2 University of Southern California Keck School of Medicine, Los Angeles, California; 3 Loyola University Health System, Maywood, Illinois; 4 Mercy Hospital & Medical Center, Chicago, Illinois

## Abstract

**Objective:**

To establish an antimicrobial stewardship program in the outpatient setting.

**Design:**

Prescribers of antimicrobials were asked to complete a survey regarding antimicrobial stewardship. We also monitored their compliance with appropriate prescribing practices, which were shared in monthly quality improvement reports.

**Setting:**

The study was performed at Loyola University Health System, an academic teaching healthcare system in a metropolitan suburban environment.

**Participants:**

Prescribers of antimicrobials across 19 primary care and 3 immediate- and urgent-care clinics.

**Methods:**

The voluntary survey was developed using SurveyMonkeyand was distributed via e-mail. Data were collected anonymously. Rates of compliance with appropriate prescribing practices were abstracted from electronic health records and assessed by 3 metrics: (1) avoidance of antibiotics in adult acute bronchitis and appropriate antibiotic treatment in (2) patients tested for pharyngitis and (3) children with upper respiratory tract infections.

**Results:**

Prescribers were highly knowledgeable about what constitutes appropriate prescribing; verified compliance rates were highly concordant with self-reported rates. Nearly all prescribers were concerned about resistance, but fewer than half believed antibiotics were overprescribed in their office. Among respondents, 74% reported intense pressure from patients to prescribe antimicrobials inappropriately. Immediate- and urgent-care prescribers had higher rates of compliance than primary-care prescribers, and the latter group responded well to monthly reports and online educational resources.

**Conclusions:**

Intense pressure from patients to prescribe antimicrobials when they are not indicated leads to overprescribing, an effect compounded by the importance of patient satisfaction scores. Compliance reporting improved the number of appropriate antibiotics prescribed in the primary care setting.

Soon after discovering penicillin, Alexander Fleming warned of the dangers associated with inappropriate use, particularly the development of resistance.^1^ Drug resistance leads to increased morbidity, mortality, and healthcare costs, and it is strongly linked to inappropriate use.^2^ One modifiable risk factor for the development of resistance is the proportion of inappropriate prescriptions for antimicrobials, especially those from outpatient healthcare facilities.^3^ Over the last 10 years, antimicrobial stewardship programs (ASPs) have made great strides in mitigating drug resistance by regulating drug use,^4^ largely with incremental improvements through quality improvement projects.^5^ However, stewardship has remained largely confined to the inpatient setting.

In 2017, the Centers for Disease Control and Prevention (CDC) estimated that 47 million outpatient antibiotic prescriptions in the United States are unnecessary,^2^ which is nearly one-third of prescriptions. In response, The Joint Commission implemented Standard MM.09.01.03 in early 2020, effectively mandating an ASP in any ambulatory organization that routinely prescribes antimicrobial medications.^6^ In anticipation of this new standard, a previously inpatient-only ASP at a suburban academic health system began to expand into the outpatient setting. We applied a behavioral intervention across our health system, similar to the Behavioral Economics to Improve Treatments of Acute Respiratory Infections (BEARI) study.^7^ Whereas individual clinicians across multiple clinics were randomized to various interventions in the BEARI trial, we applied a behavioral intervention to all outpatient prescribers throughout the entire health system.

The initial goals of the intervention included continuous evaluation, reporting, and improvement of antibiotic prescribing compliance; minimizing underutilization of antibiotics from delayed diagnoses and misdiagnoses; and ensuring proper drug, dose, and duration. To achieve these goals, a baseline survey was delivered to outpatient prescribers via e-mail, in conjunction with monitoring prescriber compliance with antimicrobial prescribing guidelines. The survey was designed to assess prescriber understanding of stewardship and antimicrobial resistance with questions modeled on the Illinois Department of Public Health (IDPH) Precious Drugs & Scary Bugs campaign.^8^ Compliance rates for prescribing habits were tracked via electronic health records and were reported to prescribers in accordance with institutional review board approval. After a year of monitoring compliance rates, institutional average and personal compliance rates were reported to each prescriber monthly. Here, we report on the impact of these interventions.

## Methods

### Study location

Loyola University Health System (LUHS) is a regional, academic health system based in Chicago’s western suburbs and a member of Trinity Health. This system includes 15 Chicago-area locations and a large ambulatory network of clinics throughout Cook, Will, and DuPage counties, offering primary and specialty care. The Infectious Disease Society of America (IDSA) designated the ASP at the main LUHS medical center an Antimicrobial Stewardship Center of Excellence in 2019.^9^

### Survey

A baseline survey was sent to outpatient physicians who prescribe antibiotics on September 27, 2019, to assess their understanding of stewardship and antimicrobial resistance (Supplementary Fig. 1). Questions were modeled from the IDPH Precious Drugs & Scary Bugs Campaign.^8^ The survey was sent to 107 prescribers at 19 primary-care and 3 immediate- or urgent-care clinics via e-mail. Prescribers were asked to participate in this survey (developed with SurveyMonkey) voluntarily and anonymously. Furthermore, 32 responses were received from September 27, 2019, until October 31, 2019 (response rate, 30%). Not all questions included in the survey are discussed here.

### Prescriber compliance

We evaluated all dispensed antibiotic prescriptions written by outpatient prescribers for rapid testing of group A *Streptococcus* and avoidance of antibiotics in patients with nonspecific upper respiratory symptoms. These metrics are based on quality improvement measurements assembled in the Healthcare Effectiveness Data and Information Set (HEDIS) and informed by the IDSA.^2^ Compliance rates were tracked from December 2018 through August 2021 and reported to prescribers in a monthly e-mail (Supplementary Fig. 2A and 2B).

The compliance rates for these 3 metrics were reported to all prescribers (Supplementary Fig. 3A and 3B) for the overall health system and each prescriber. For these compliance rates, the denominator is the number of total prescriptions fitting diagnosis codes for bronchitis, pharyngitis, or upper respiratory infection (URI) from encounters with an antibiotic prescribed and the numerator is the number of prescriptions that were appropriate according to quality measurements assembled in the HEDIS.

### Electronic education material

In February 2020 all primary care providers received an educational presentation in PowerPoint (Microsoft, Redmond, WA) attached to that month’s compliance report e-mail, providing online education about appropriate antibiotics use (Supplementary Fig. 4). Prescribers were encouraged (not required) to view the educational material.

### Study approval

The survey was conducted with the consent of the local IRB under protocol no. 212212. Prescriber compliance with antimicrobial prescribing data was collected as part of an ongoing quality improvement project at LUHS.

### Statistics

With a categorical predictor variable (before versus after the intervention) and 2 quantitative outcome variables (percent compliance), we used the unpaired *t* test for testing how significant the difference was between the groups.

## Results

### Survey and respondent information

In total, 107 physician prescribers of antimicrobials who were affiliated with the Loyola University Health System (LUHS) were asked to voluntarily complete an anonymous survey online. Although the anonymity of the survey allowed us to encourage more forthcoming and earnest evaluations without concern for reprisal and retaliation, this may be seen as a limitation to the study. Furthermore, 32 prescribers responded to the request and completed the voluntary survey. Respondents worked in primary care clinics (38% adults; 34% adults and pediatrics) or immediate/urgent care clinics (25%), with 6% electing to not disclose.

### Knowledge and perception of antimicrobial stewardship and resistance

**Fig. 1.**
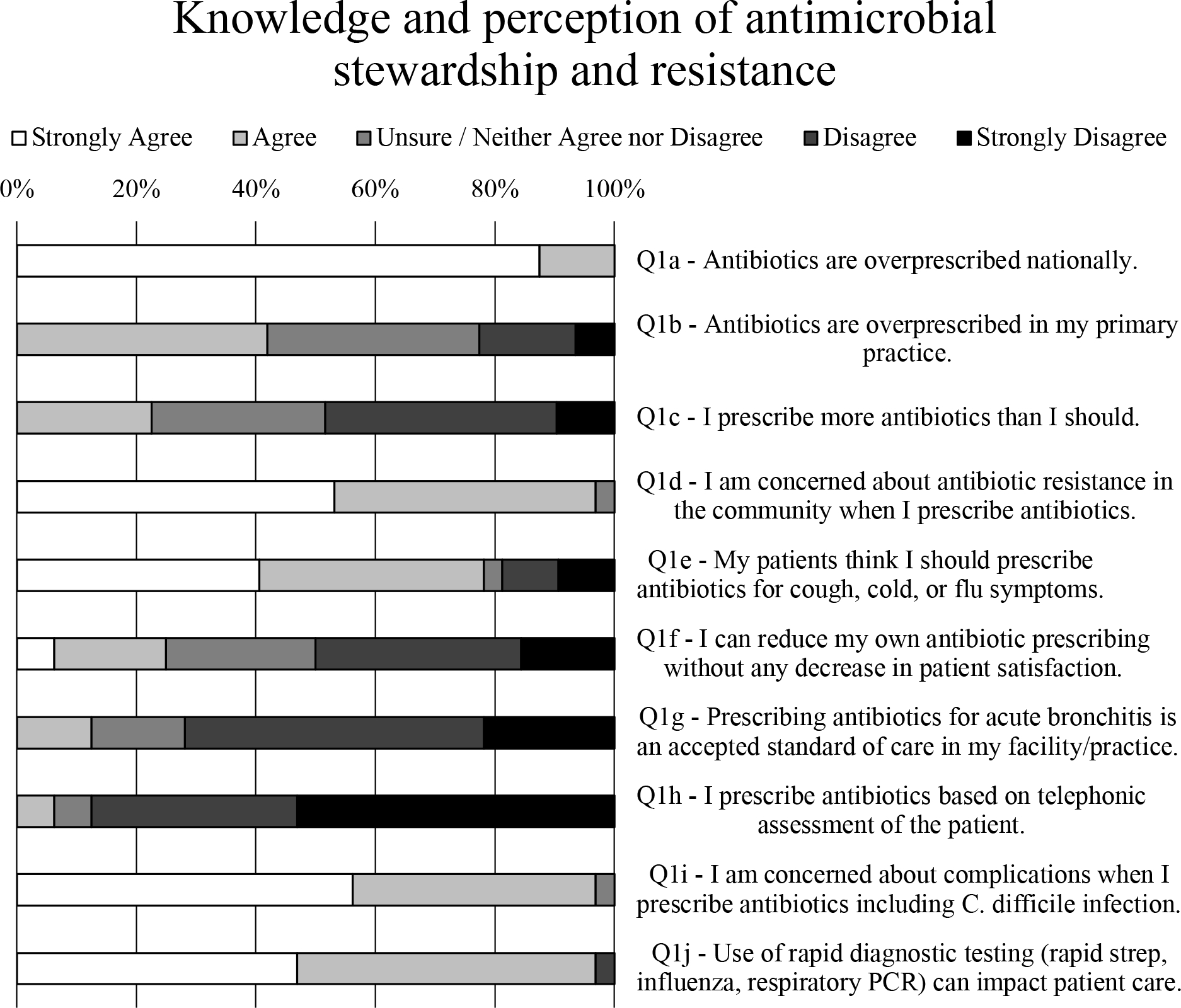
Survey data for prescriber knowledge and perception of antimicrobial stewardship and resistance.

Respondents were highly knowledgeable about antibiotics being overprescribed nationally (Fig. 1, Q1a) but were less convinced that this phenomenon was occurring in their own practice (Fig. 1, Q1b). Indeed, respondents were twice as likely to disagree than agree when asked whether they prescribed more antibiotics than they should (Fig. 1, Q1c). They were 6 times more likely to disagree than agree that prescribing antibiotics for acute bronchitis was an accepted standard of care in their facility or practice (Fig. 1, Q1g). Nevertheless, almost all respondents (97%) expressed concern about antibiotic resistance in their community (Fig. 1, Q1d) as well as complications from using antibiotics, including *Clostridioides difficile* infection (Fig. 1, Q1i).

When asked whether they could reduce their own antibiotic prescribing without any decrease in patient satisfaction, twice as many respondents felt unable to do so compared to those who felt they could (Fig. 1, Q1f). This finding may be the result of most prescribers (78%) reporting that their patients believe they should be prescribed antibiotics for cough, cold, or flu symptoms (Fig. 1, Q1e). One respondent stated that “patients can be REALLY pushy—and sometimes I can get enough [history] by phone to be comfortable, but usually not.” Another said, “The biggest challenge from an immediate- or urgent-care perspective is when our evidence-based practices do not match what patients are used to receiving from their PCPs” (primary care physicians) and recounted patients who encounter prescribers who provide them with antibiotics on demand. Yet another prescriber explained, “Patients often initially want antibiotics for viral infections, though are often teachable and not demanding.”

Putting these responses in the context of COVID-19, 88% of respondents to our prepandemic survey overwhelmingly disagreed rather than agreed (6%) that they prescribe antibiotics based on telephonic assessment of the patient (Fig. 1, Q1h). Nearly all (97%) felt that use of rapid diagnostic testing [eg, rapid strep, influenza, or respiratory polymerase chain reaction (PCR)] can influence patient care (Fig. 1, Q1j).

### Provider prescription of antibiotics

**Fig. 2.**
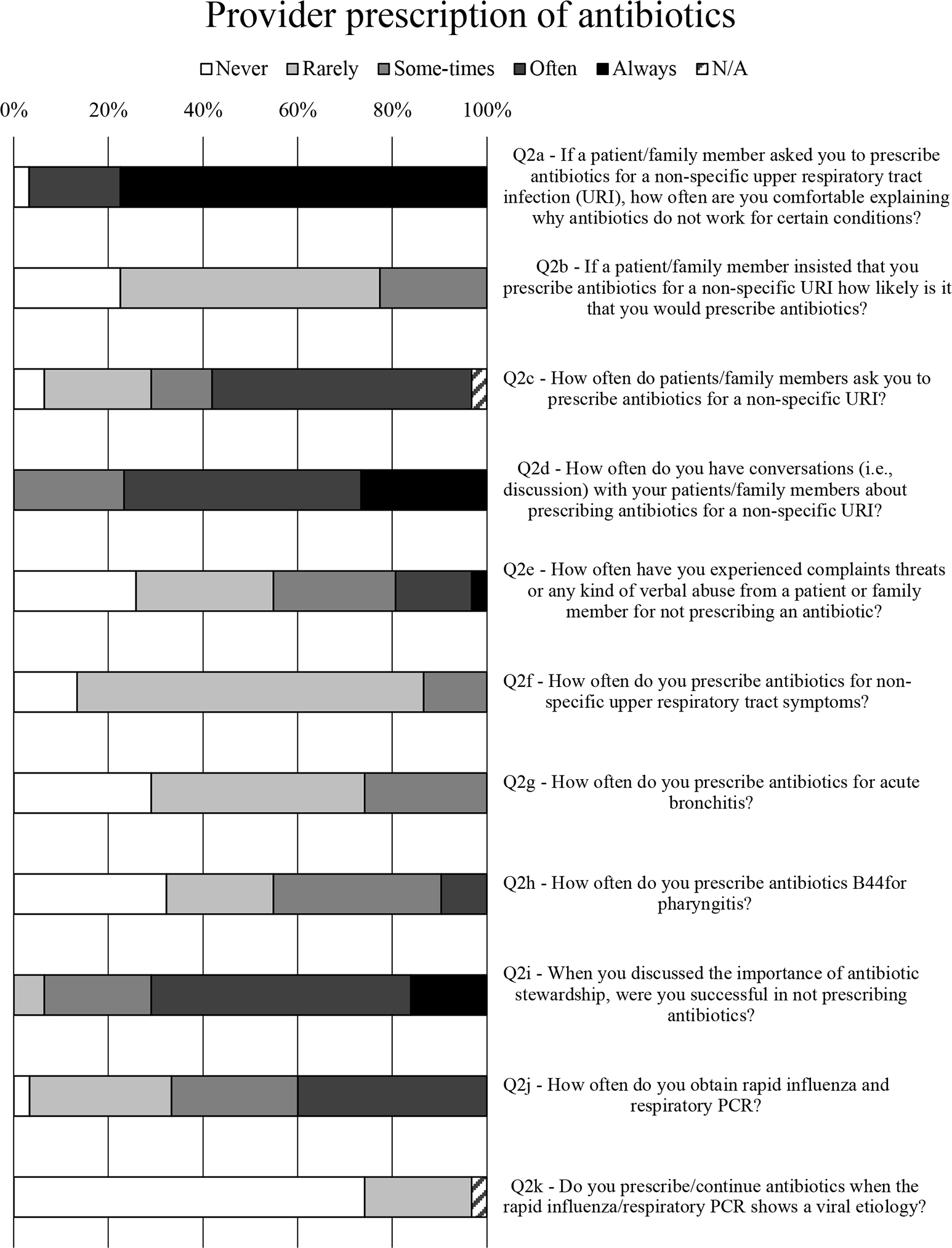
Survey data for provider antibiotic prescribing habits.

Overall, 97% of respondents felt comfortable explaining why antibiotics do not work for certain conditions (Fig. 2, Q2a). Also, 90% were asked to prescribe antibiotics for a nonspecific URI (Fig. 2, Q2c), and all indicated that they would have a conversation about prescribing antibiotics for a nonspecific URI (Fig. 2, Q2d). Three-quarters of respondents received complaints, threats, or verbal abuse for not prescribing an antibiotic (Fig. 2, Q2e). When asked how often they prescribed antibiotics for a nonspecific URI, most (86%) would never or rarely prescribe antibiotics for a nonspecific URI (Fig. 2, Q2f), but this rate dropped to 77% if the patient insisted on an antibiotic prescription (Fig. 2, Q2b). Still, the weighted average of responses was exactly the same (ie, 2.00 or “rarely”), regardless of whether asked this question in the context of an insistent patient (Fig. 2, Q2b) or in general (Fig. 2, Q2f).

For acute bronchitis, 74% of respondents would rarely or never prescribe antibiotics (Fig. 2, Q2g). For pharyngitis, 55% of prescribers indicated that they would rarely or never prescribe antibiotics, and another 33% only sometimes prescribed antibiotics (Fig. 2, Q2h). When discussing the importance of stewardship, all prescribers indicated at least some level of success, and 71% indicated that they were often or always successful in not prescribing antibiotics (Fig. 2, Q2i).

One respondent recounted having a “patient complain to patient relations when ‘all she did was tell me to have soup, tea, Tylenol, and a hot shower.’ ” Almost all prescribers (97%) indicated that they used rapid influenza and respiratory PCR tests (Fig. 2, Q2j). However, one prescriber commented, “I don’t need a rapid influenza/respiratory PCR to know that a viral infection is viral and doesn’t need antibiotics.” Another lamented that they “would love to use rapid flu or resp (sic) PCR, but PCR is cost prohibitive.” Upon confirmation of viral etiology via rapid influenza or respiratory PCR test, 23% of prescribers indicated that they rarely prescribed/continued antibiotics, although 77% indicated that they never did (Fig. 2, Q2k).

### Factors influencing antibiotic prescribing for acute URI of uncertain etiology

**Fig. 3.**
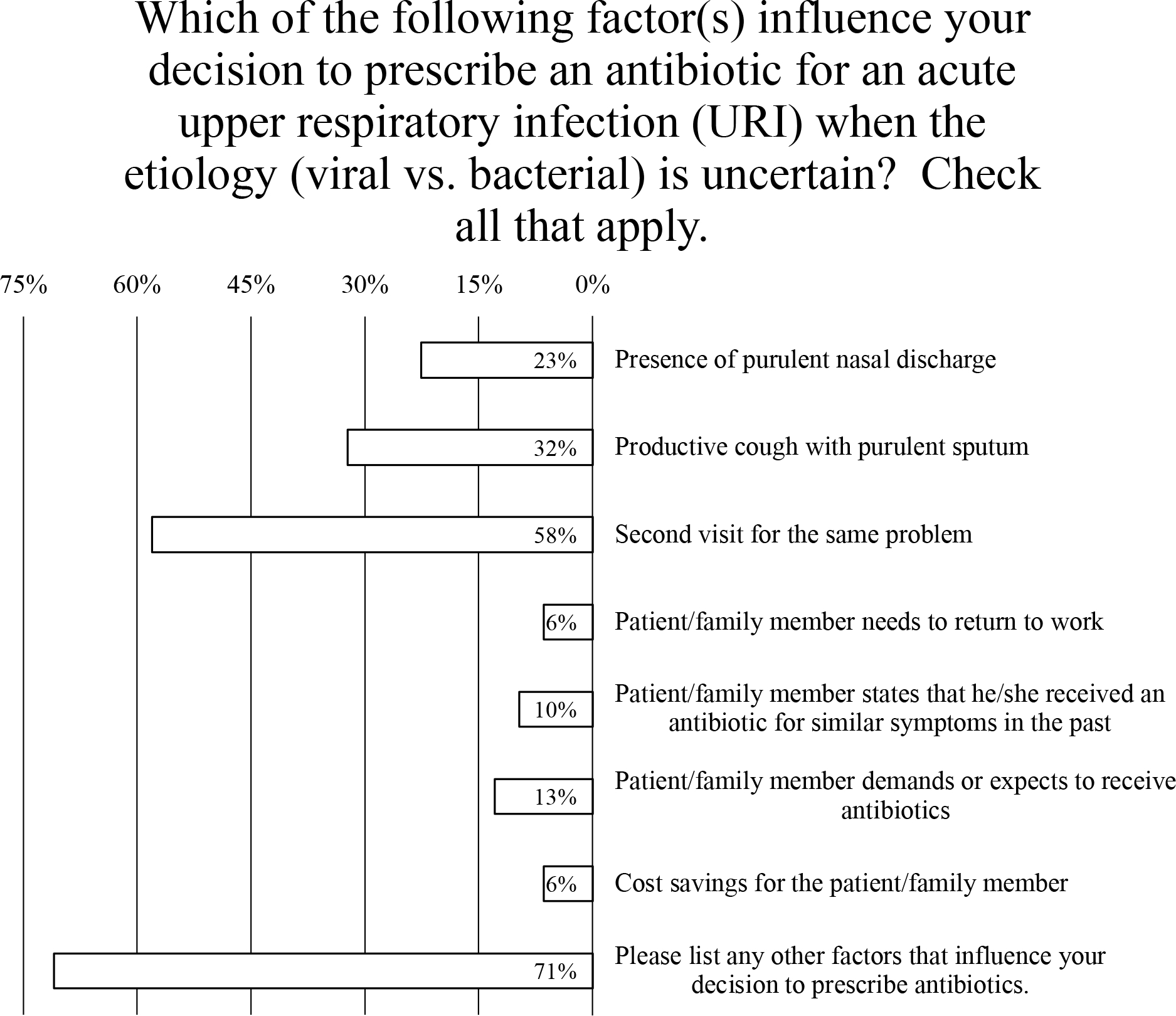
Survey data for factors influencing the decision to prescribe an antibiotic.

When patients present with an acute URI, the 3 most influential factors for deciding whether to prescribe an antibiotic were second visit for the same problem (endorsed by 58% of prescribers), productive cough with purulent sputum (endorsed by 32% of prescribers), and presence of purulent nasal discharge (endorsed by 23% of prescribers) (Fig. 3). Less important to prescribers were the patient’s need to return to work (endorsed by 6% of prescribers), cost savings for patient (endorsed by 6% of prescribers), receipt of antibiotic for similar symptoms in the past (endorsed by 10% of prescribers), and patient demanding or expecting to receive antibiotics (endorsed by 13% of prescribers) (Fig. 3). Also, 60% of respondents’ comments indicated that the “length of illness” or “duration of symptoms” influenced their decision to prescribe antibiotics (Fig. 3). Furthermore, 27% of respondents also commented that “comorbidities such as COPD,” a “history of asthma,” and “immune status” played a role in their decision to prescribe antibiotics (Fig. 3).

### Delayed Prescribing

**Fig. 4.**
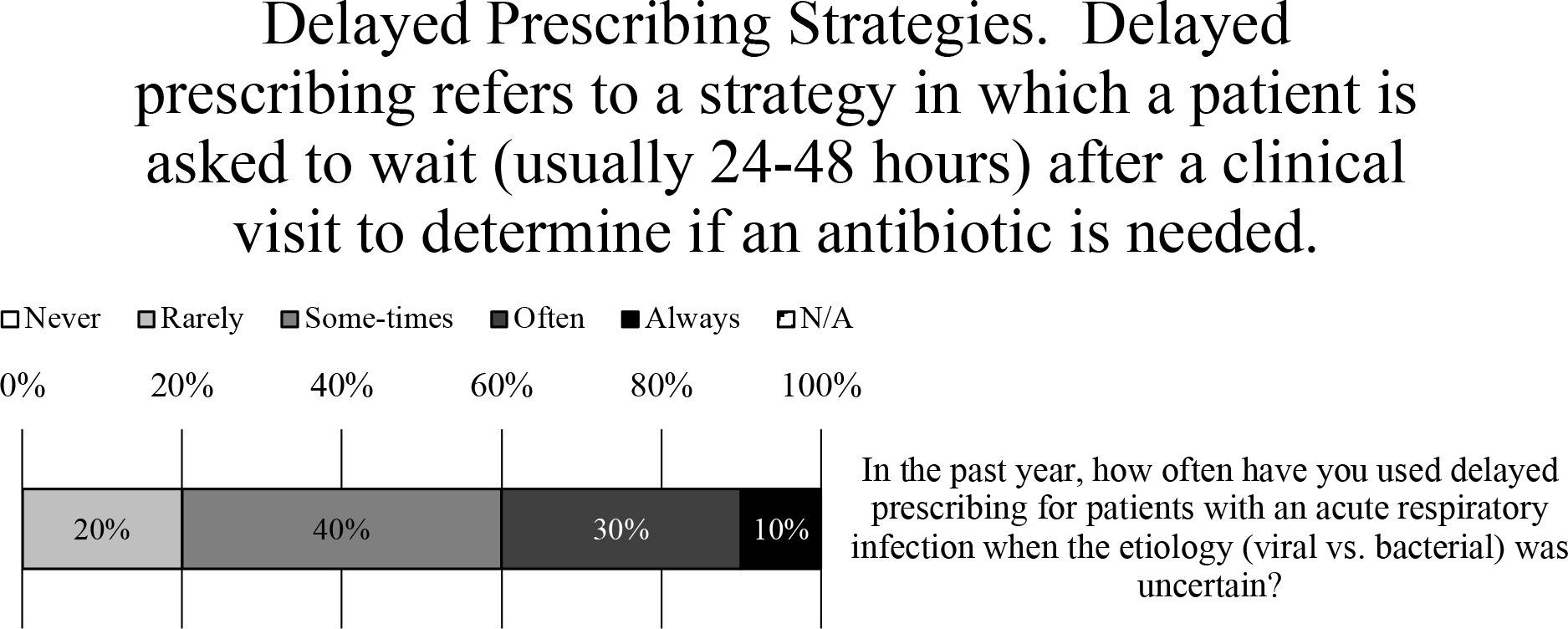

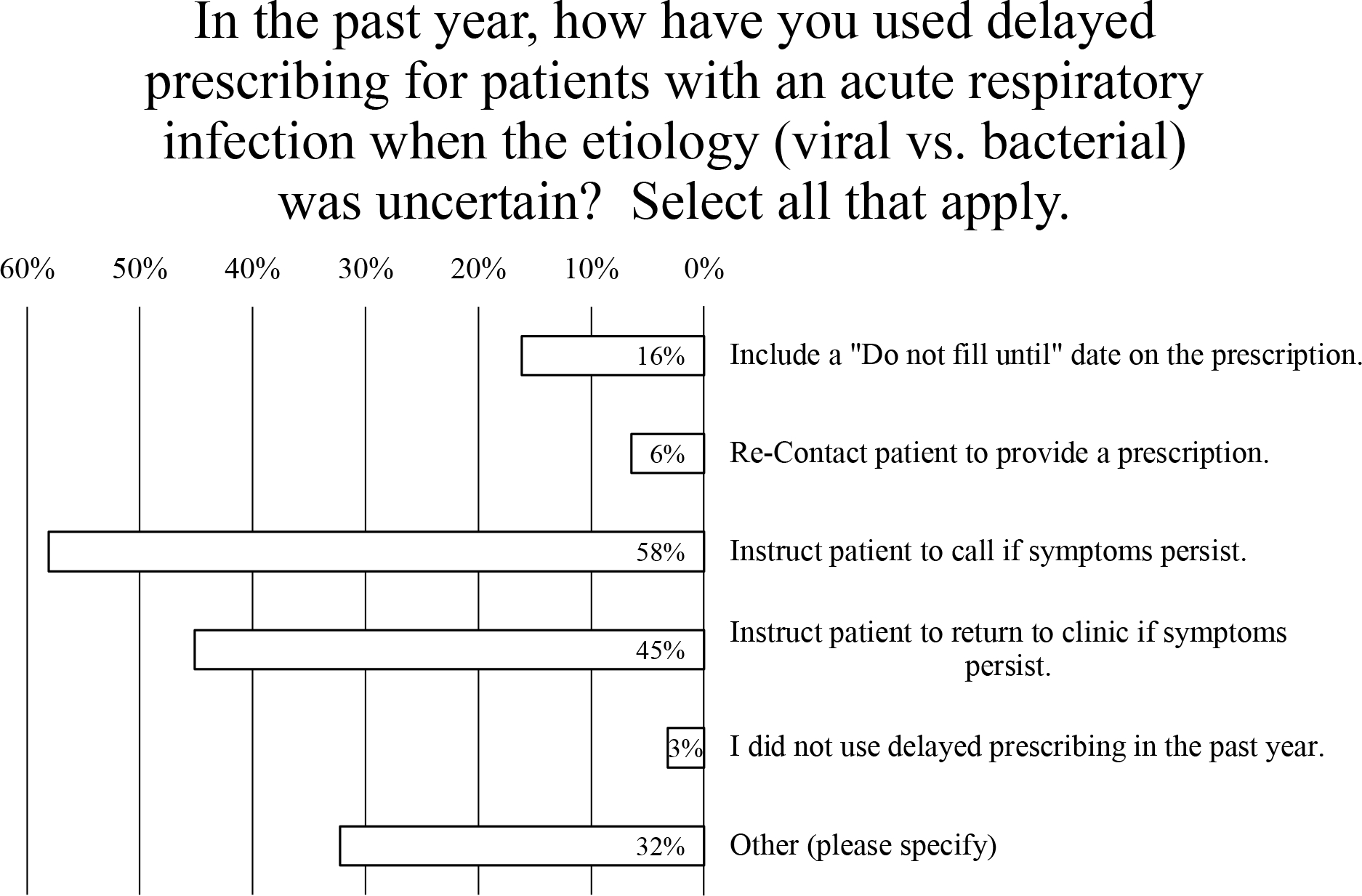
Delayed Prescribing Practices. (A) Survey data for frequency of using delayed prescribing strategies. (B) Survey data for type of delayed prescribing strategies used.

After a clinic visit fails to reveal the etiology (ie, viral or bacterial) of an acute URI, nearly all prescribers (97%) indicated that they used delayed prescribing for patients (Fig. 4B). When asked how frequently, the weighted average of responses was between “sometimes” and “often,” with 10% marking “always” and 30% marking “often” (Fig. 4A). One prescriber explained, “This is my go-to strategy as a PCP—make patients aware I am easy to access should things worsen” (Fig. 4A). Another said they use delayed prescribing strategies “almost always for acute otitis in children” (Fig. 4A).

When asked how they have used delayed prescribing, nearly all the most common methods were to instruct patients to call if their symptoms persist (endorsed by 56% of prescribers) or to return to the clinic if their symptoms persist (endorsed by 44% of prescribers) (Fig. 4B). Less frequently employed were including a “do not fill until” date on the prescription (endorsed by 16% of prescribers) or recontacting patient to provide prescription (endorsed by 6% of prescribers) (Fig. 4B). Also, 80% of comments (26% of prescribers) noted that they would write a prescription for antibiotics and “instruct” or “recommend” the patient not fill it unless their symptoms do not improve after “48 hours” or “3–5 days” or “10–14 days” after the initial visit (Fig. 4B).

### Patient education

**Fig. 5.**
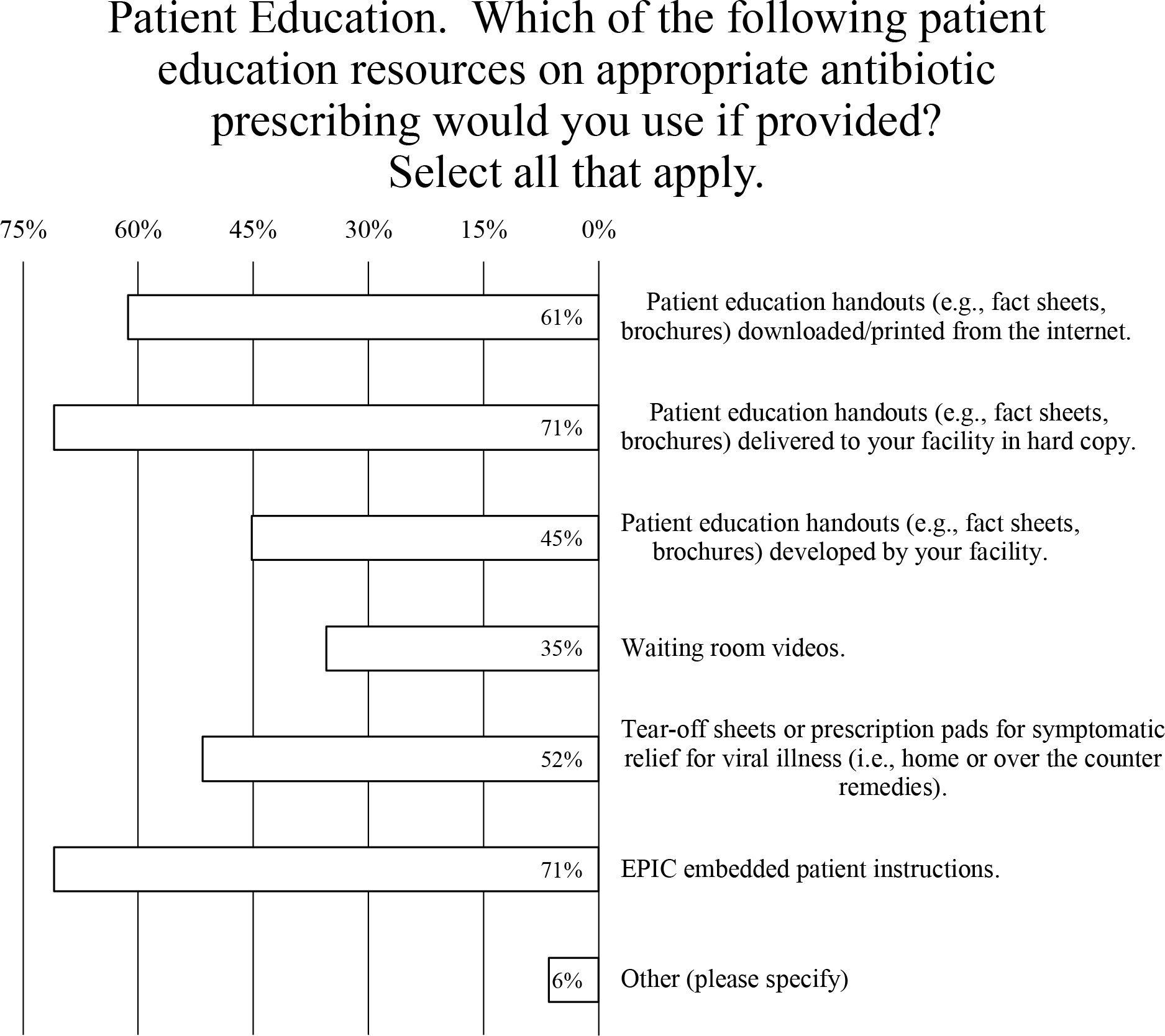

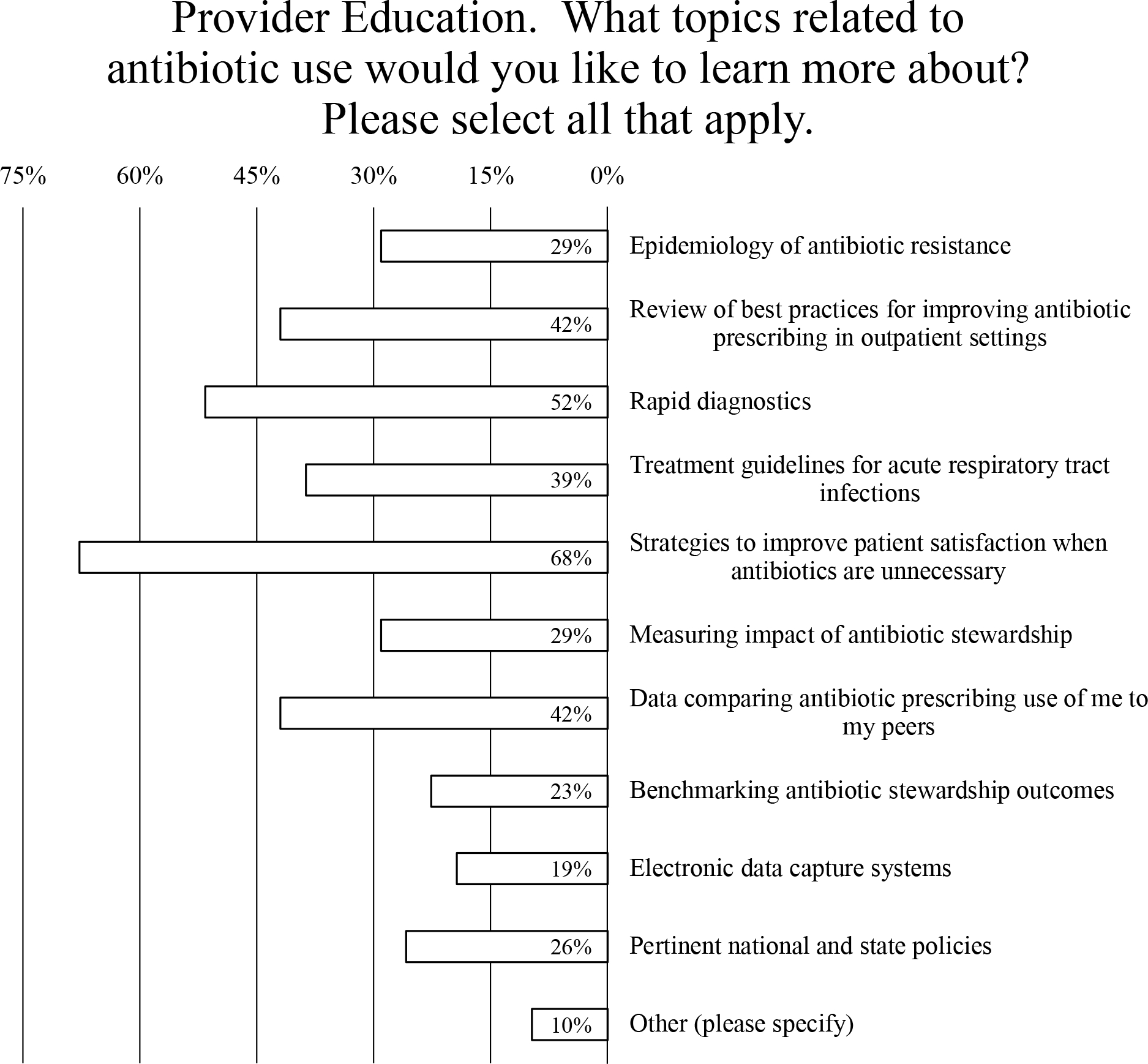
Survey data for type of antibiotic educational resources that prescribers would like (A) to provide to patients and (B) to obtain for themselves.

Most physicians (71%) indicated that, if provided, they would use patient education handouts delivered to their facility in hard copy or if embedded in their electronic health record (EHR) software (Fig. 5A). Similarly, most respondents (61%) would use such handouts if they could be downloaded or printed from the Internet (Fig. 5A). Somewhat less popular were the use of prescription pads for home or over-the-counter remedies (endorsed by 52% of prescribers), patient education handouts developed by their facility (endorsed by 45% of prescribers), and waiting-room videos (endorsed by 35% of prescribers) (Fig. 5A).

### Provider education

Prescribers were most interested in learning about strategies to improve patient satisfaction when antibiotics are unnecessary (endorsed by 68% of prescribers) (Fig. 5B). Rapid diagnostics were also highly valued (endorsed by 52% of prescribers), more than reviewing best practices for outpatient antibiotics prescribing and data comparing antibiotic prescribing use to the prescriber’s peers (both endorsed by 42% of prescribers), as well as treatment guidelines for acute respiratory tract infections (endorsed by 39% of prescribers) (Fig. 5B). Less interest was expressed for learning about the epidemiology of antibiotic resistance or measuring the impact of antibiotic stewardship (both endorsed by 29% of prescribers) (Fig. 5B). Even less popular were learning about pertinent national and state policies (endorsed by 26% of prescribers), benchmarking antibiotic stewardship outcomes (endorsed by 23% of prescribers), and electronic data capture systems (endorsed by 19% of prescribers) (Fig. 5B).

### Prescriber compliance

**Fig. 6.**
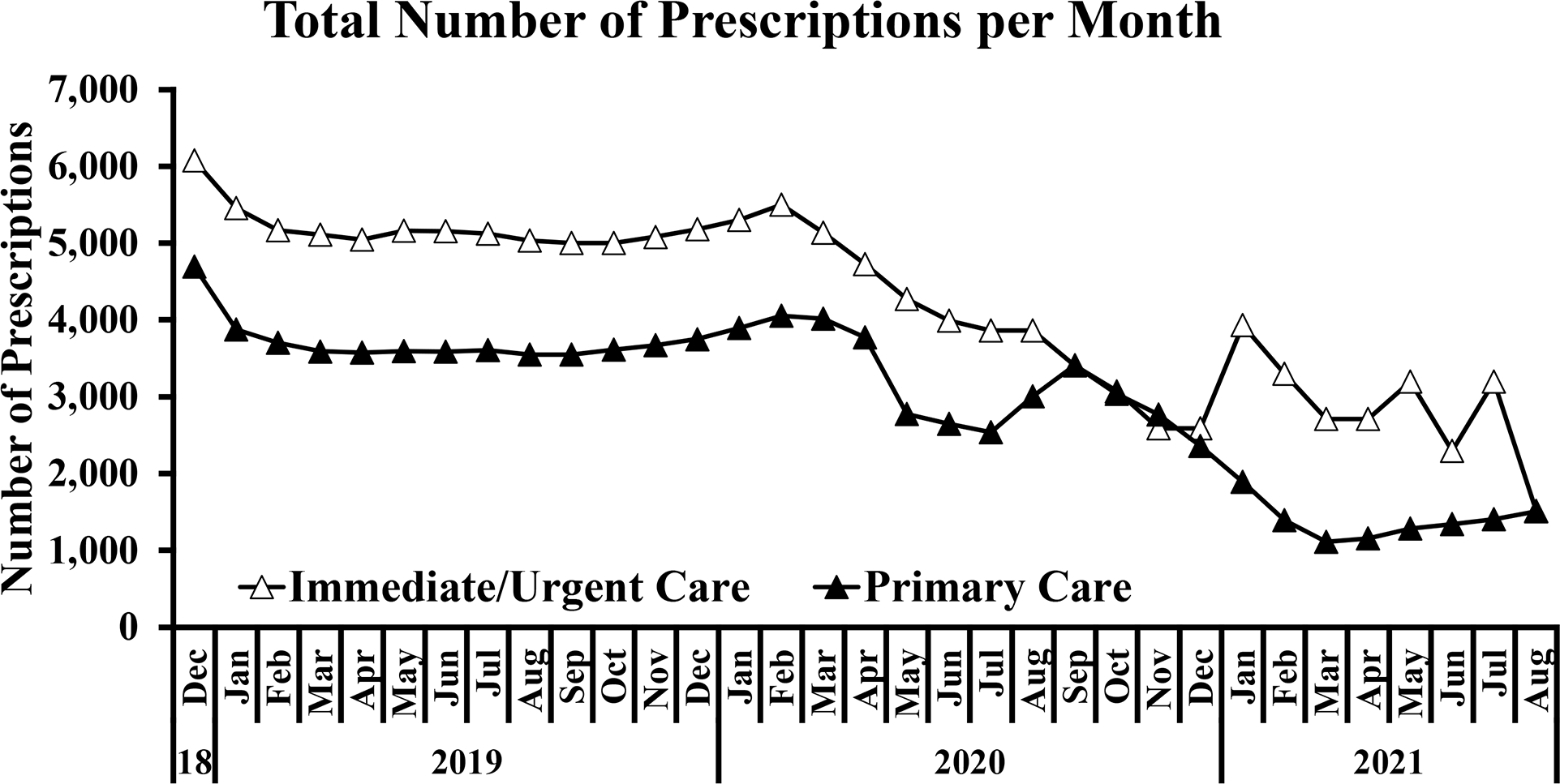

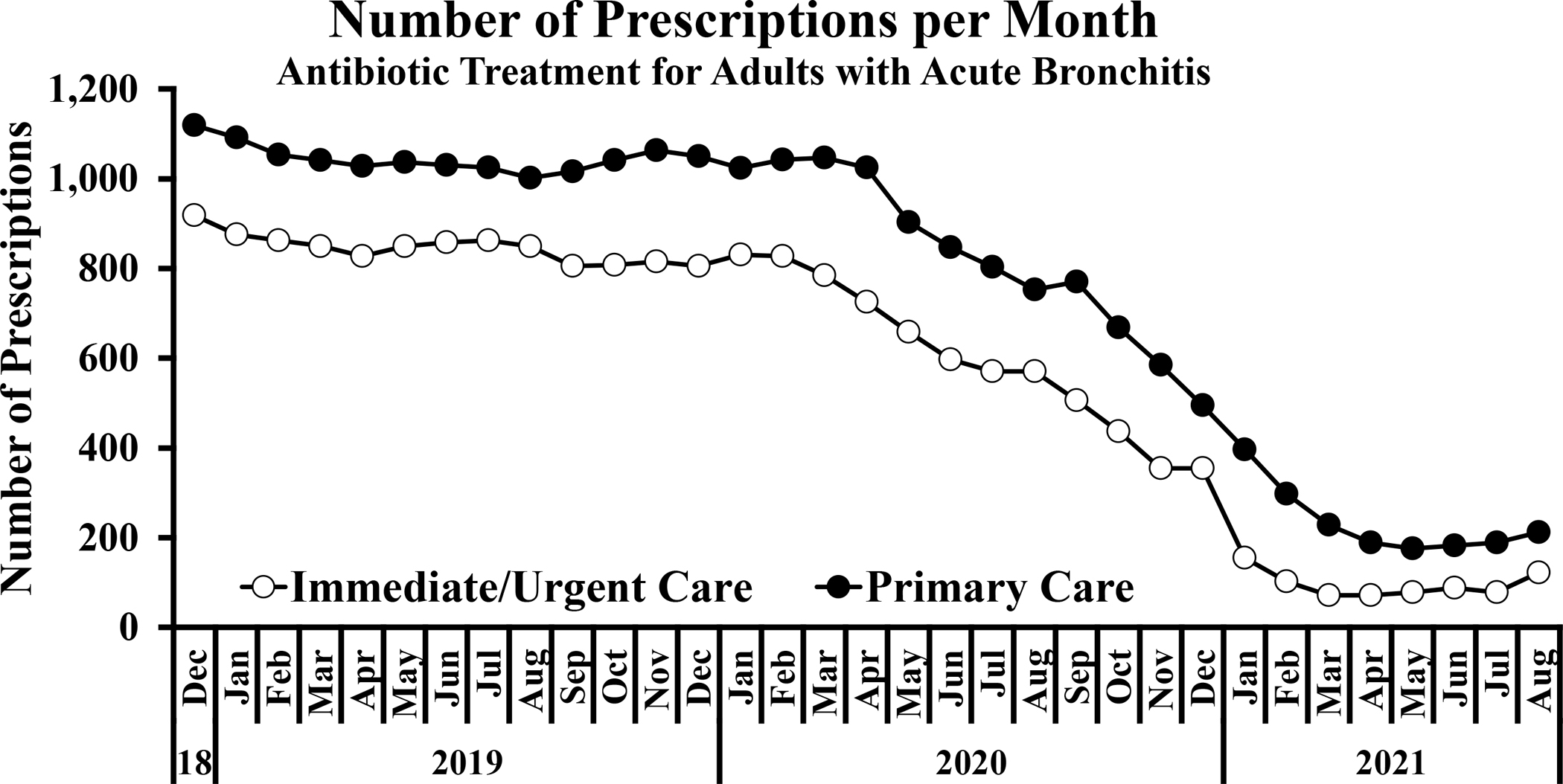

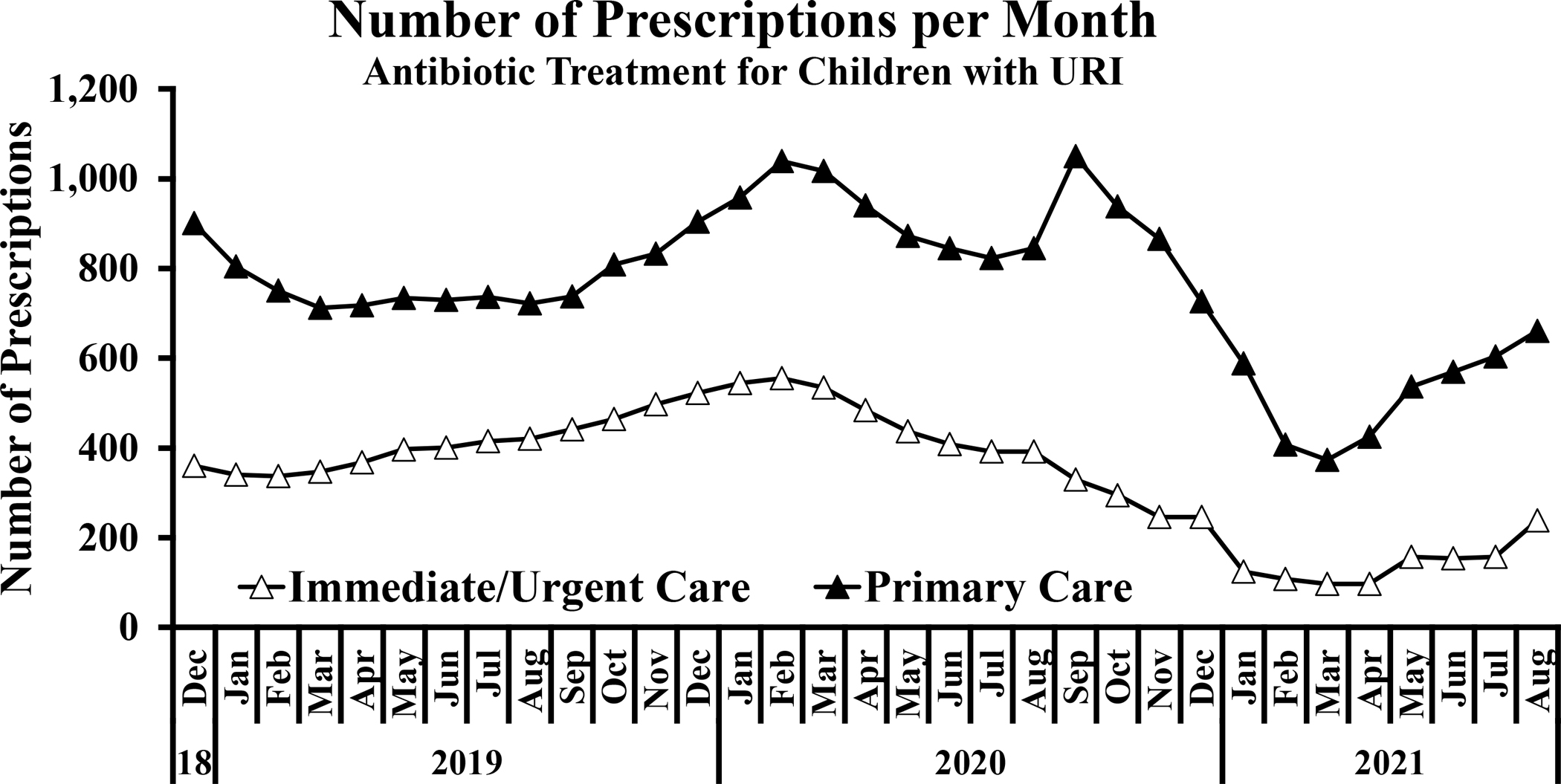

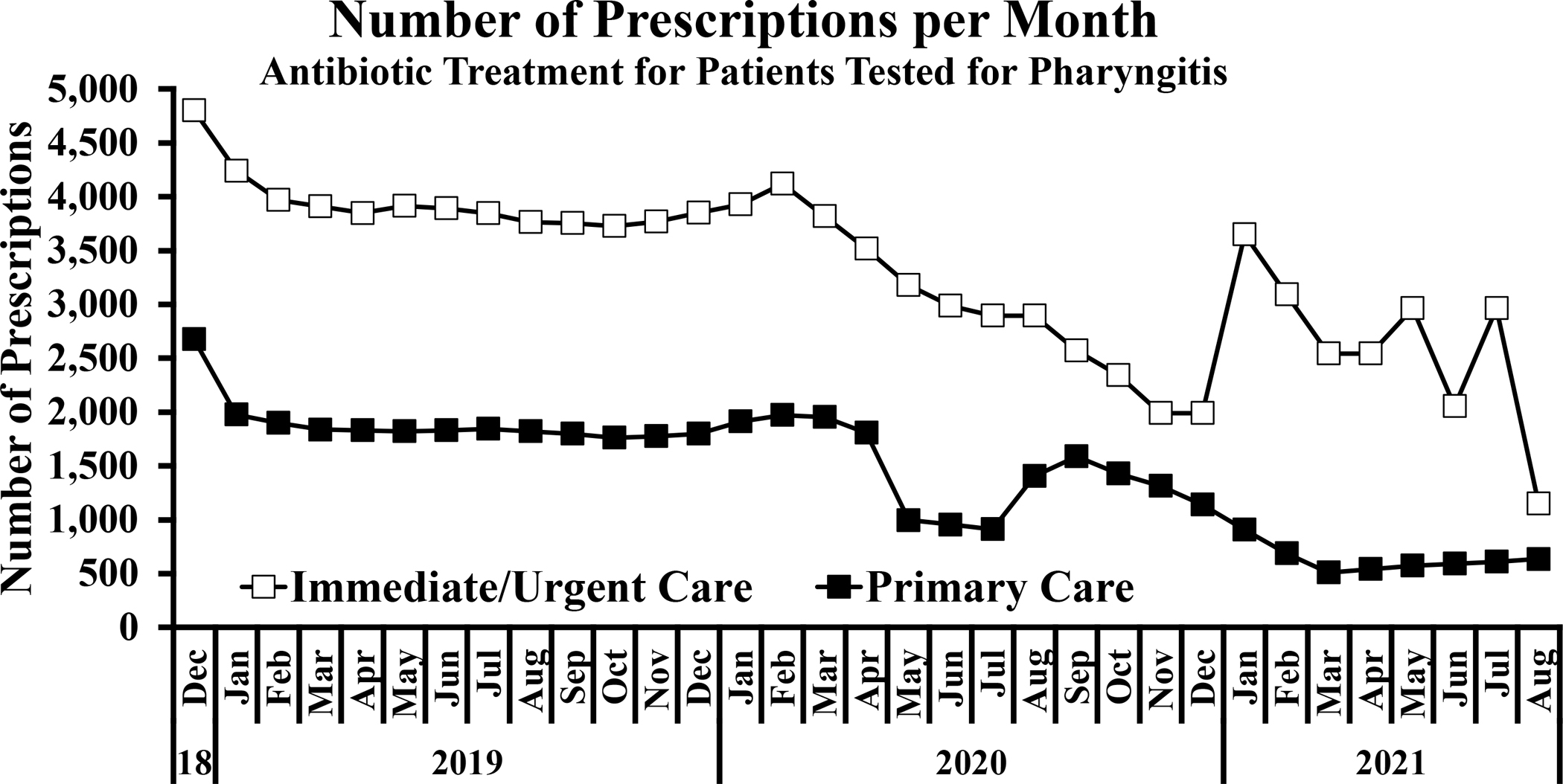
Antibiotics prescriptions per month. The number of prescriptions remained relatively stable across 2019, dropped during 2020, and stabilized again in 2021, particularly when considering (A) the total number of prescriptions and (B) antibiotic treatment for adults with acute bronchitis. This trend also applied to the number of prescriptions for (C) antibiotic treatment for children with URI and (D) antibiotic treatment for patients tested for pharyngitis.

**Fig. 7.**
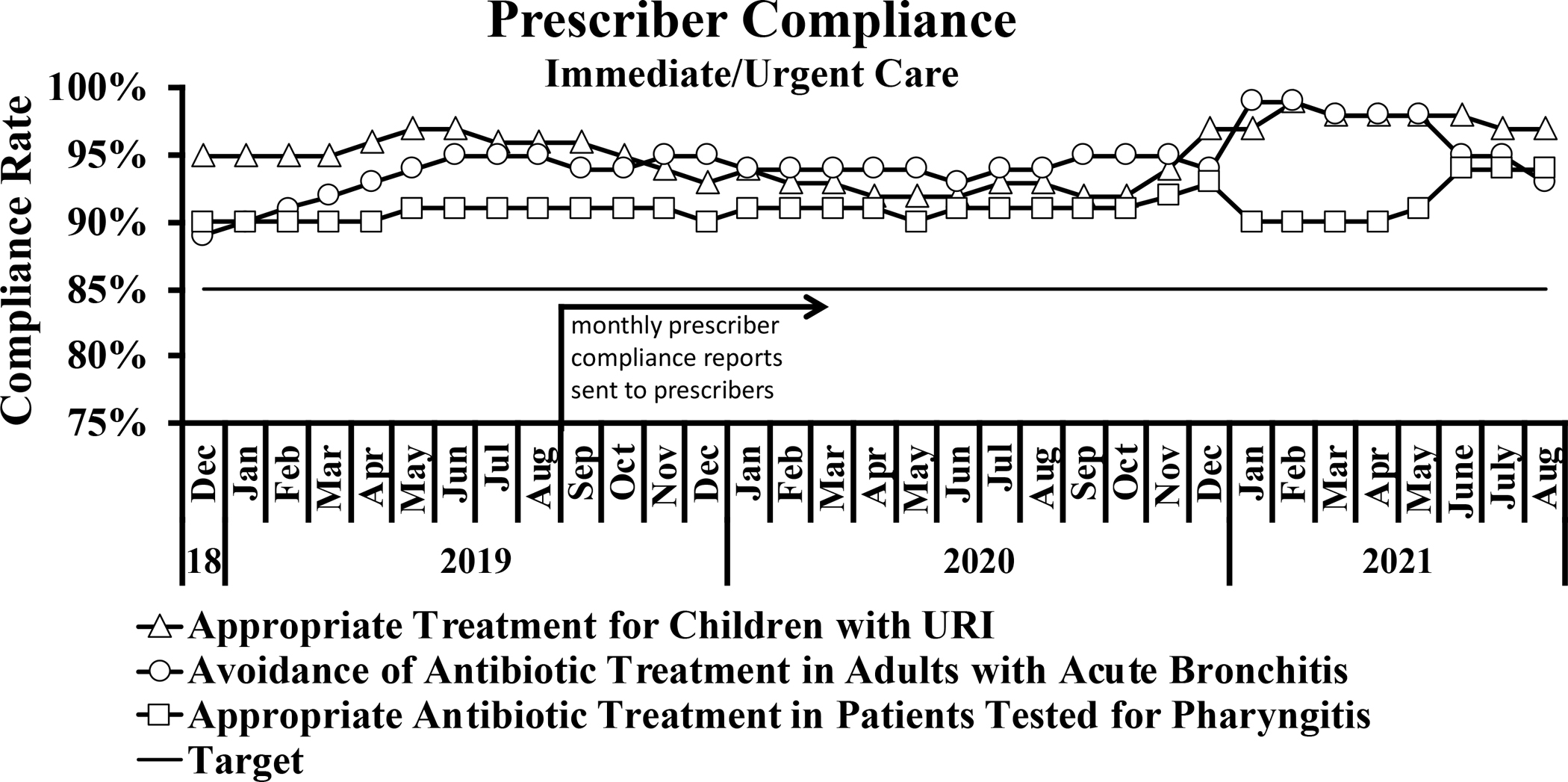

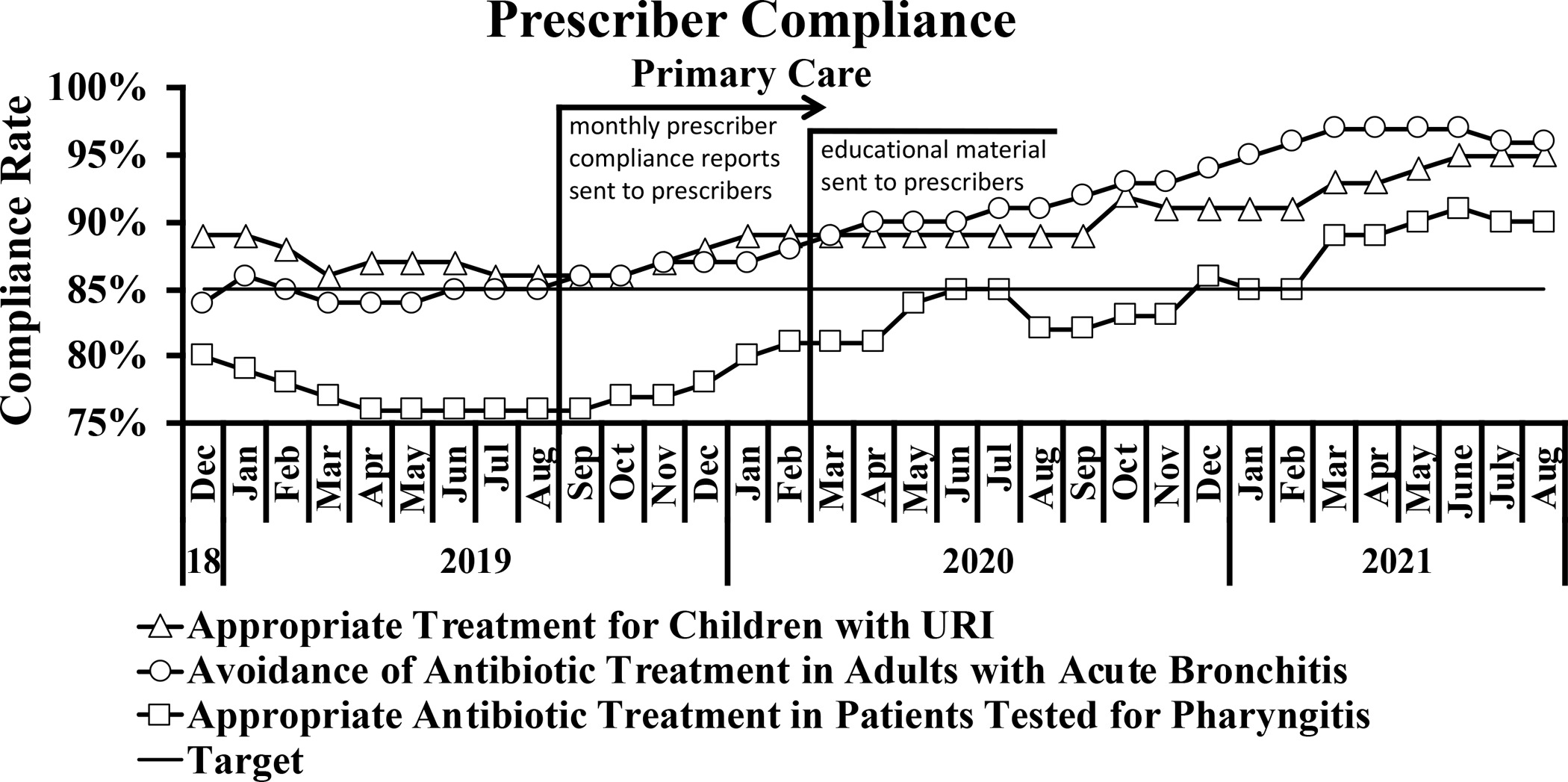
Prescriber compliance rates. (A) Compliance rates for immediate- or urgent-care prescribers were above the target threshold for all 3 metrics assessed. For monthly reporting before and after the intervention, *P* = .3071 for appropriate treatment for children with URI, *P* = .0007 for avoidance of antibiotic treatment in adults with acute bronchitis, and *P* = .0805 for appropriate antibiotic treatment in patients tested for pharyngitis. (B) Primary care prescribers responded well to compliance reports and education. For monthly reporting before and after the intervention, *P* = .0021 for appropriate treatment for children with URI, *P* = .0001 for avoidance of antibiotic treatment in adults with acute bronchitis, and *P* = .0001 for appropriate antibiotic treatment in patients tested for pharyngitis. For electronic education material before and after initiating monthly reporting, *P* = .0010 for appropriate treatment for children with URI, *P* = .0001 for avoidance of antibiotic treatment in adults with acute bronchitis, and *P* = .0002 for appropriate antibiotic treatment in patients tested for pharyngitis.

Reports were created using data abstracted from the EHR to show prescriber compliance rate with antibiotics prescribing guidelines. During the study, antibiotic prescribing peaked in December 2018 and reached its nadir in March 2021 for Primary Care and August 2021 for Immediate/Urgent Care (Fig. 6A). The total number of prescriptions for all 3 quality measures remained relatively stable for 2019, decreased throughout 2020, and stabilized at a new low rate in 2021 (Fig. 6A). This was particularly the case with antibiotic treatment for adults with acute bronchitis (Fig. 6B) and to a lesser extent with antibiotic treatment for children with URI (Fig. 6C) and for patients tested for pharyngitis (Fig. 6D). At the time of the survey, all 3 prescriber compliance rates were well below 100%, and the primary care clinics—but not immediate or urgent care clinics—were below the targeted rate of 85% (Fig. 7A and 7B). Still, these compliance rates were highly concordant with the anonymously recorded self-reported rates. Compliance rates were tracked from December 2018 through August 2021 to allow for comparison before and after the intervention (Fig. 7A and 7B).

Immediate- and urgent-care prescribers had already attained high rates of compliance prior to receiving monthly reports, yet there was a statistically significant (*P* = .0007) minor improvement (3% increase) in compliance for avoiding antibiotic treatment in adults with acute bronchitis (Fig. 7A). Primary care prescribers had lower baseline compliance rates and responded well to monthly reports and an educational e-mail, which highlighted certain survey results and reinforced IDSA *Streptococcus* pharyngitis guidelines (Fig. 7B). All 3 measures yielded statistically significant improvements in compliance both after initiating the monthly compliance reporting as well as the electronic education material (.0001 ≤ *P* ≤ .0021).

## Discussion

Intense pressure from patients to prescribe antimicrobials when they are not indicated can lead to overprescribing. This effect is compounded by the importance placed upon patient satisfaction scores by the Centers for Medicare & Medicaid Services (CMS).^10^ The results of the current study underscore the difficulty of withholding antibiotics from patients. Despite there being no clinical benefit to delaying the prescription of antibiotics for patients like those investigated in this study,^11^ nearly all survey respondents (97%) indicated that they use some form of delayed prescribing (Fig. 4B). Indeed, delaying prescriptions is much more likely to result in the use of antibiotics than no prescription at first encounter, but delayed prescriptions have been shown to improve patient satisfaction ratings associated with not prescribing antibiotics at first encounter.^11^

One prescriber commented, “The ‘need’ to return to work is not one I take lightly. Many of my patients are paid hourly wages, aren’t allowed sick leave, and are struggling to pay bills. I am sympathetic to their need to ‘get well fast.’ ” Additionally, King et al^12^ found that some patients seek a diagnosis, and if it is confirmed to be viral, would be more willing to forgo antibiotics. Nevertheless, detecting viral etiology in the outpatient setting does not change the treatment plan and introduces excess costs.^13^

In the current study, reporting compliance rates to prescribers of antibiotics was associated with reducing inappropriate antimicrobial therapy in our primary-care settings, in line with what others have shown.^14^ However, the survey data reinforce the importance of behavioral interventions to bolster ASP efficacy in the outpatient setting. One prescriber commented that their former employer “utilized EPIC embedded antibiotic prescribing best practices and peer quality reports related to prescribing habits,” and strongly supported their use at LUHS. Similarly, 71% prescribers indicated that they would use patient education if embedded in the EHR software (Fig. 5A). Unfortunately, reluctance to introducing such advanced features into EHR software across institutions is pervasive.^15^ One prescriber commented, “Current EHRs were not created to support . . . improved care quality and population health management. . . . Our healthcare system has mostly not rewarded these activities. They have not been mission critical for providers or, therefore, EHR designers.”^16^

Notably, this study took place during the onset of the coronavirus disease 2019 (COVID-19) pandemic. We noticed an inverse correlation between the implementation of COVID-19 restrictions and the quantity of antibiotics prescribed per month at our clinics (Fig. 6A). This change reflects the national trend of substantially decreased antibiotics prescribing, more than would be expected from seasonal trends alone,^17^ complicating the evaluation of improved prescriber compliance rates.

Going forward, we plan to conspicuously exhibit posters in exam rooms, indicating institutional commitment to the enumerated ASP guidelines. This behavioral intervention has been shown to work and is inexpensive, simple, and effective for modifying the behavior of prescribers and patient expectations.^12,18^ Many governmental public health agencies recommend their use, including the IDPH.^8^ Future studies will allow for comparison of compliance rates before and after introducing these commitment posters, in addition to changes in prescriber understanding of stewardship and antimicrobial resistance.

## Supplementary Material

Supplemental Figure 1

Supplemental Figure 2A

Supplemental Figure 2B

Supplemental Figure 3A

Supplemental Figure 3B

Supplemental Figure 4
