# Supplementary material for: Introducing antimicrobial stewardship to the outpatient clinics of a suburban academic health system
Source: Antimicrob Steward Healthc Epidemiol. 2022 Jan 17;2(1):e9. doi: 10.1017/ash.2021.228 (PMC9319114; doi:10.1017/ash.2021.228)
Supplement: Supplementary file 1 [file S2732494X2100228Xsup001.pptx]

## Slide 1
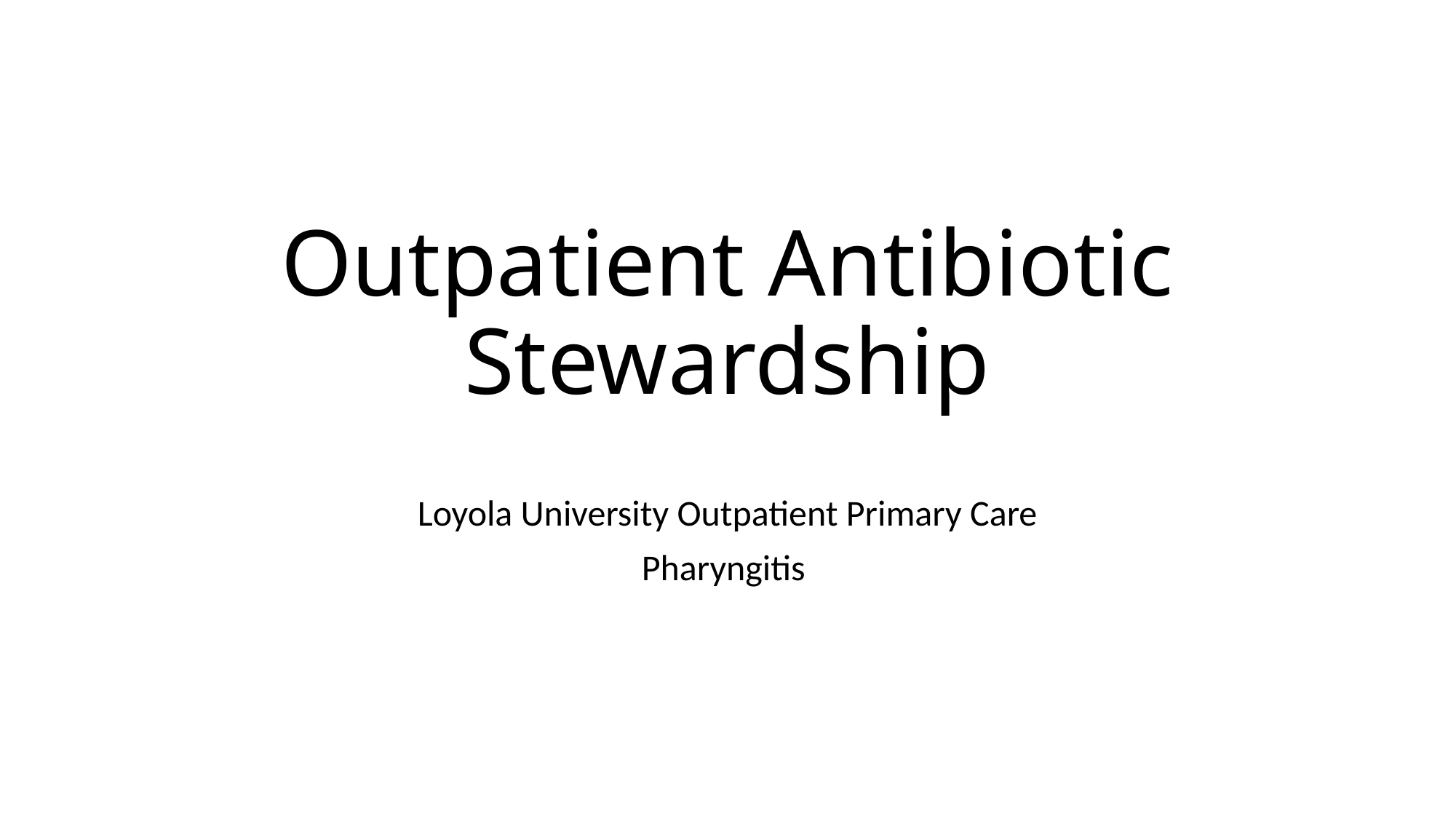

# Outpatient Antibiotic Stewardship
Loyola University Outpatient Primary Care
Pharyngitis

## Slide 2
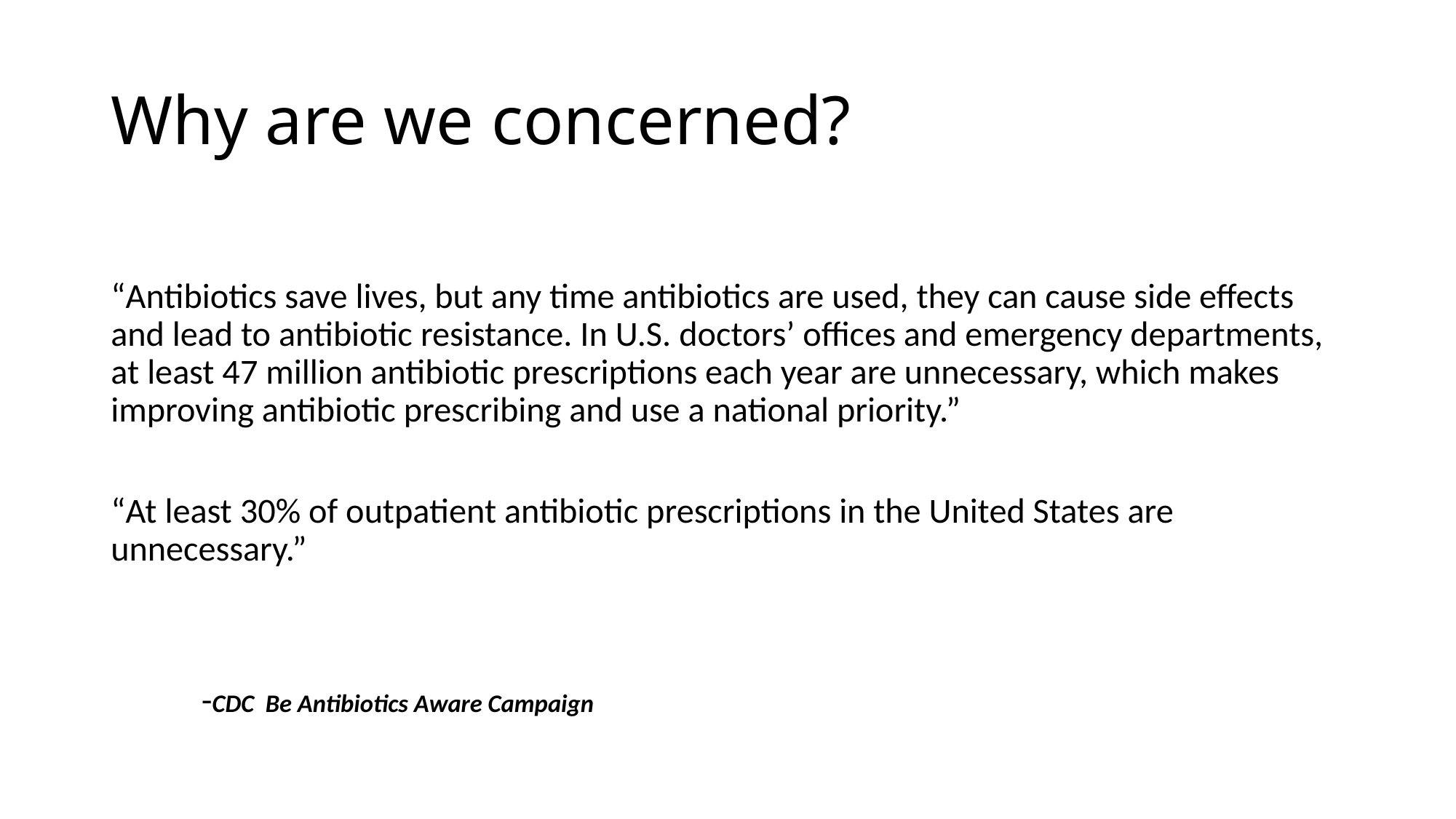

# Why are we concerned?
“Antibiotics save lives, but any time antibiotics are used, they can cause side effects and lead to antibiotic resistance. In U.S. doctors’ offices and emergency departments, at least 47 million antibiotic prescriptions each year are unnecessary, which makes improving antibiotic prescribing and use a national priority.”
“At least 30% of outpatient antibiotic prescriptions in the United States are unnecessary.”
						-CDC Be Antibiotics Aware Campaign

## Slide 3
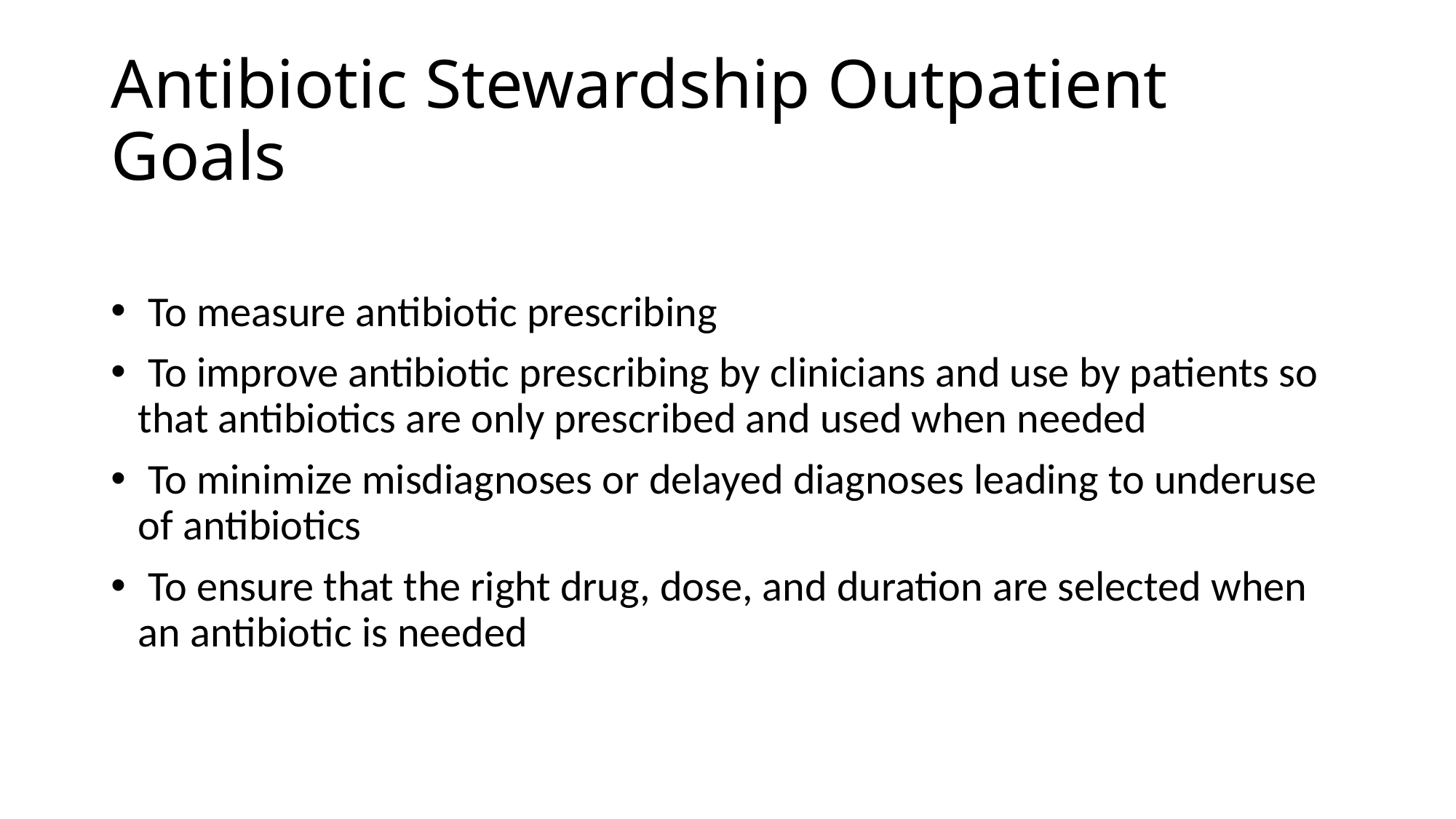

# Antibiotic Stewardship Outpatient Goals
 To measure antibiotic prescribing
 To improve antibiotic prescribing by clinicians and use by patients so that antibiotics are only prescribed and used when needed
 To minimize misdiagnoses or delayed diagnoses leading to underuse of antibiotics
 To ensure that the right drug, dose, and duration are selected when an antibiotic is needed

## Slide 4
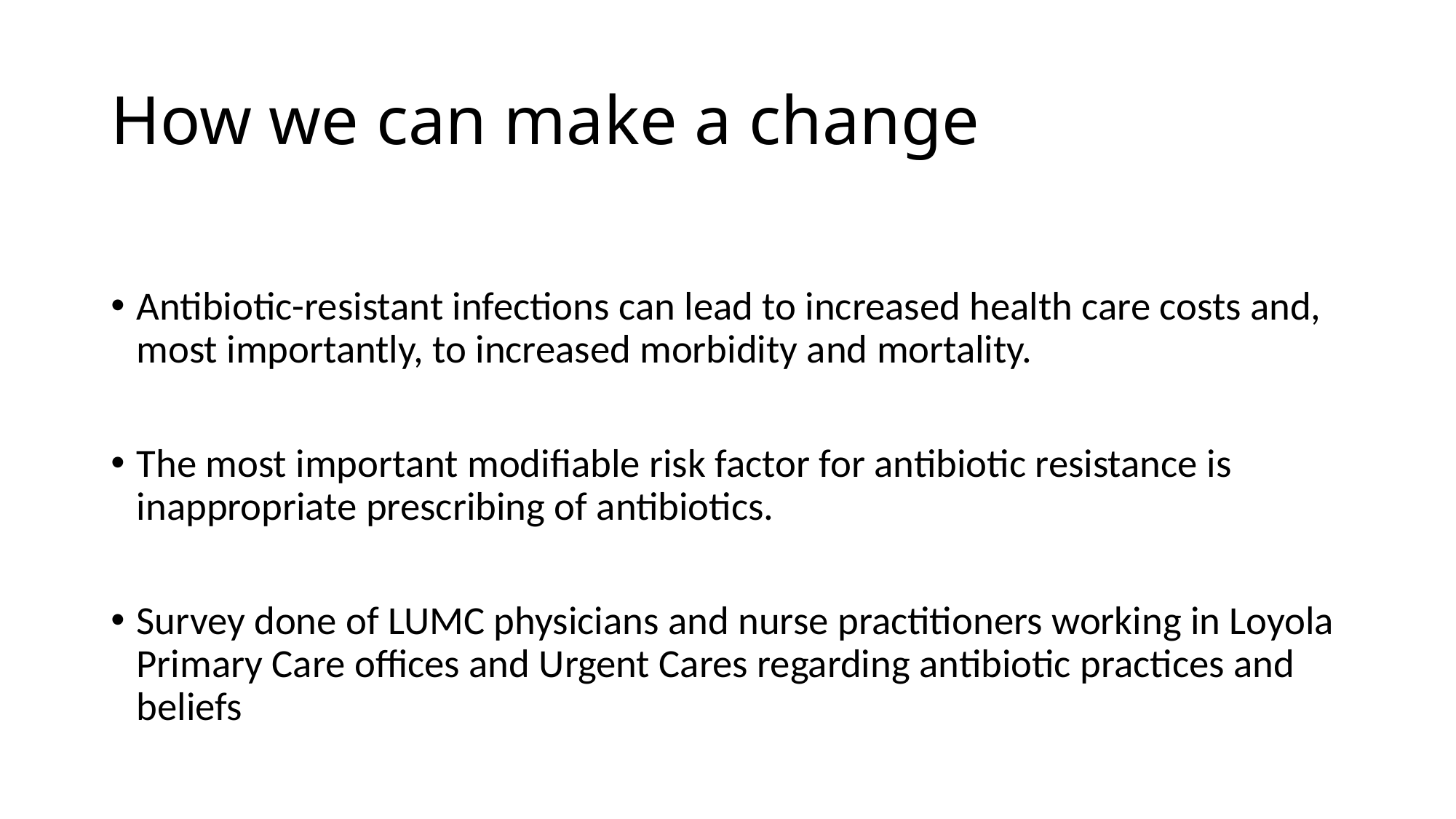

# How we can make a change
Antibiotic-resistant infections can lead to increased health care costs and, most importantly, to increased morbidity and mortality.
The most important modifiable risk factor for antibiotic resistance is inappropriate prescribing of antibiotics.
Survey done of LUMC physicians and nurse practitioners working in Loyola Primary Care offices and Urgent Cares regarding antibiotic practices and beliefs

## Slide 5
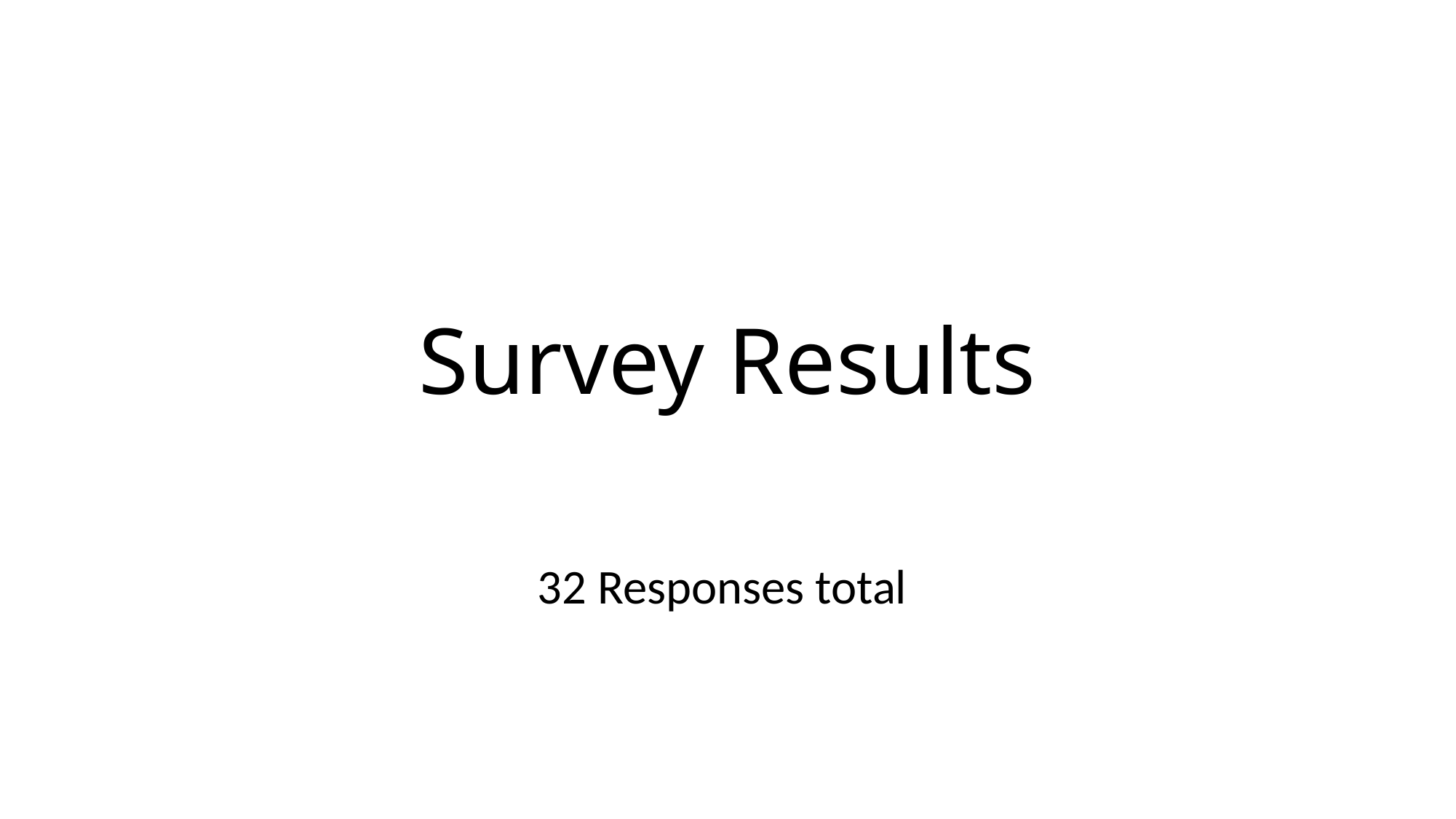

# Survey Results
32 Responses total

## Slide 6
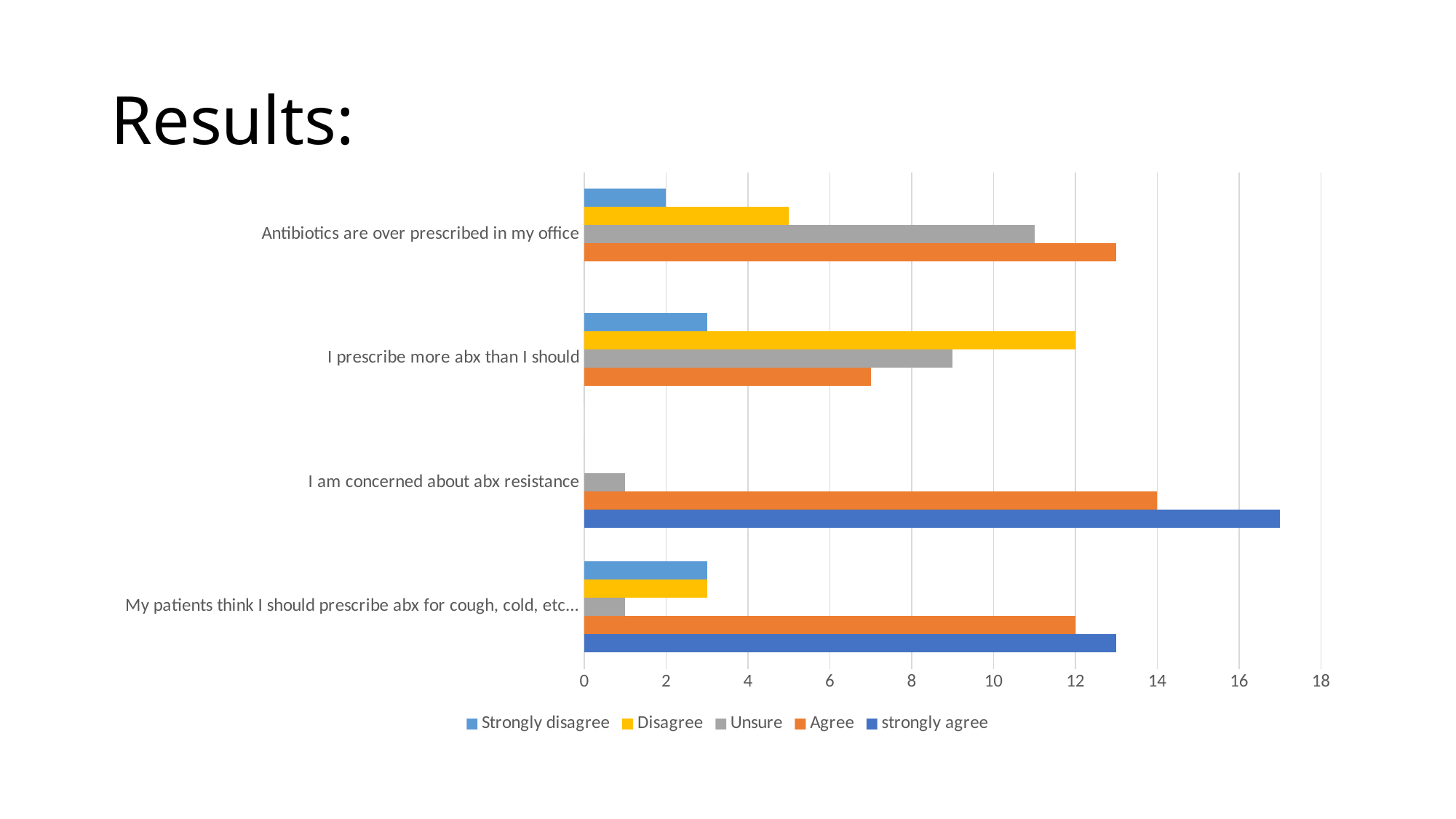

# Results:
### Chart
| Category | strongly agree | Agree | Unsure | Disagree | Strongly disagree |
|---|---|---|---|---|---|
| My patients think I should prescribe abx for cough, cold, etc... | 13.0 | 12.0 | 1.0 | 3.0 | 3.0 |
| I am concerned about abx resistance | 17.0 | 14.0 | 1.0 | 0.0 | 0.0 |
| I prescribe more abx than I should | 0.0 | 7.0 | 9.0 | 12.0 | 3.0 |
| Antibiotics are over prescribed in my office | 0.0 | 13.0 | 11.0 | 5.0 | 2.0 |

## Slide 7
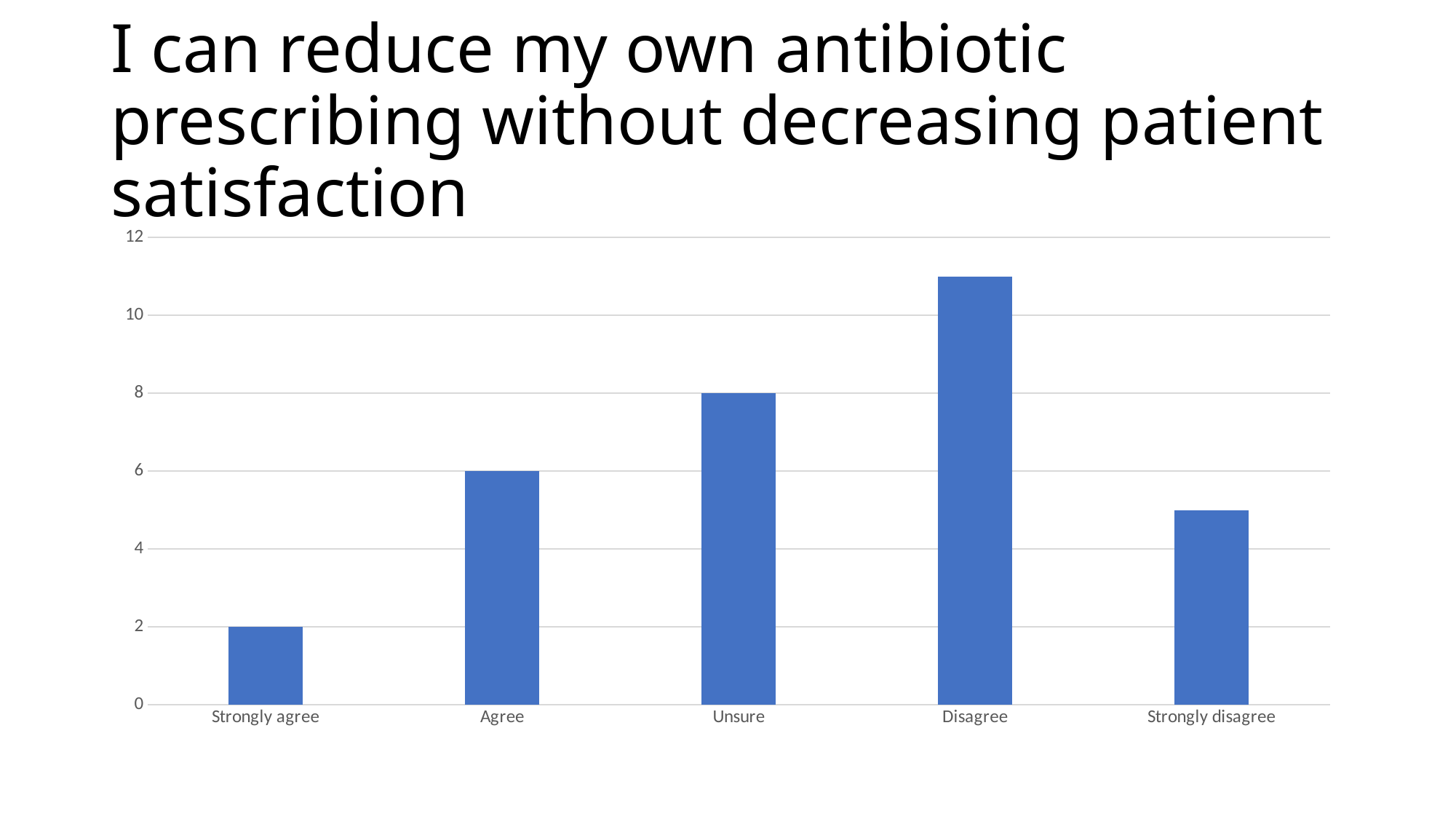

# I can reduce my own antibiotic prescribing without decreasing patient satisfaction
### Chart
| Category | Series 1 |
|---|---|
| Strongly agree | 2.0 |
| Agree | 6.0 |
| Unsure | 8.0 |
| Disagree | 11.0 |
| Strongly disagree | 5.0 |

## Slide 8
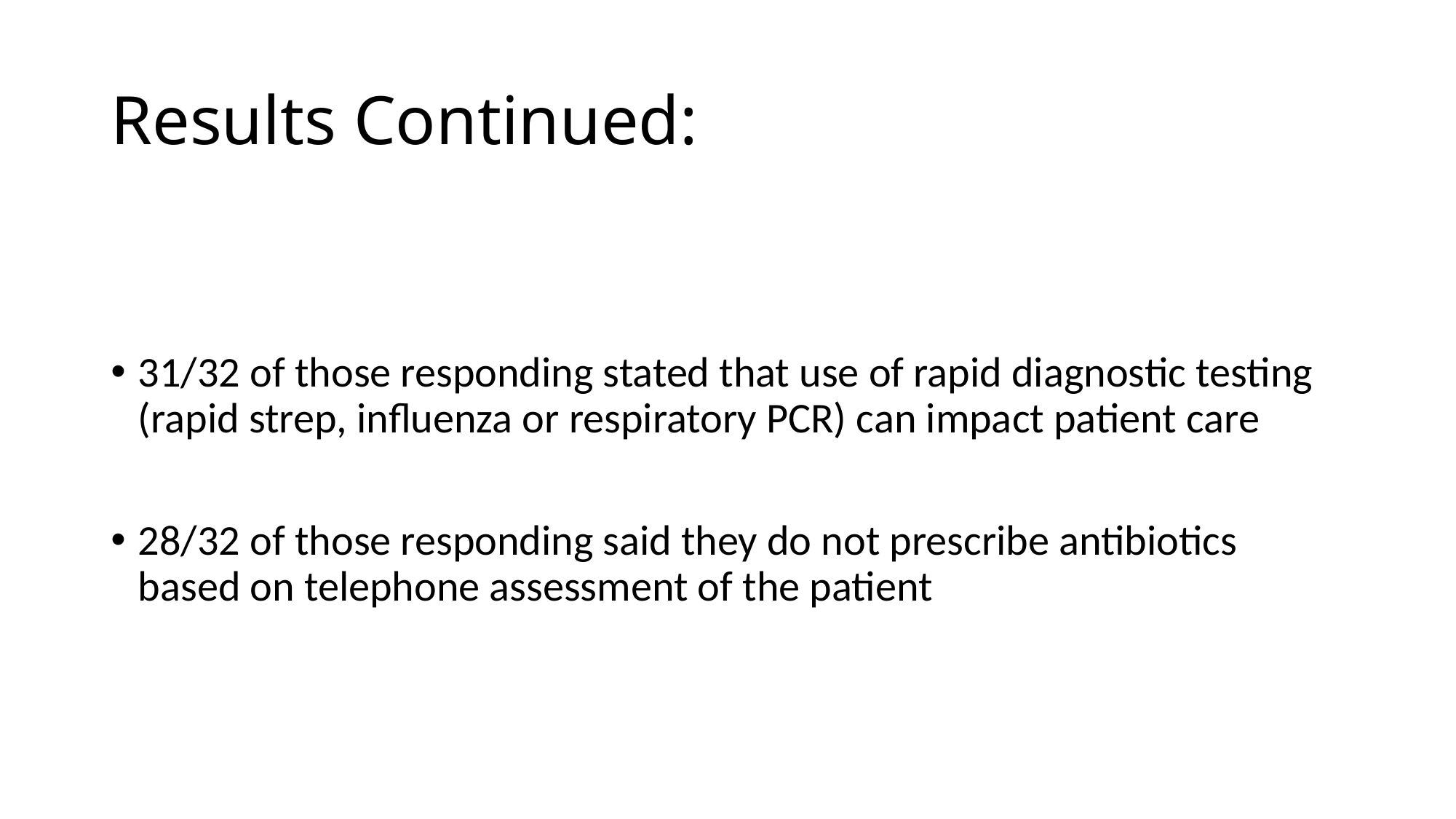

# Results Continued:
31/32 of those responding stated that use of rapid diagnostic testing (rapid strep, influenza or respiratory PCR) can impact patient care
28/32 of those responding said they do not prescribe antibiotics based on telephone assessment of the patient

## Slide 9
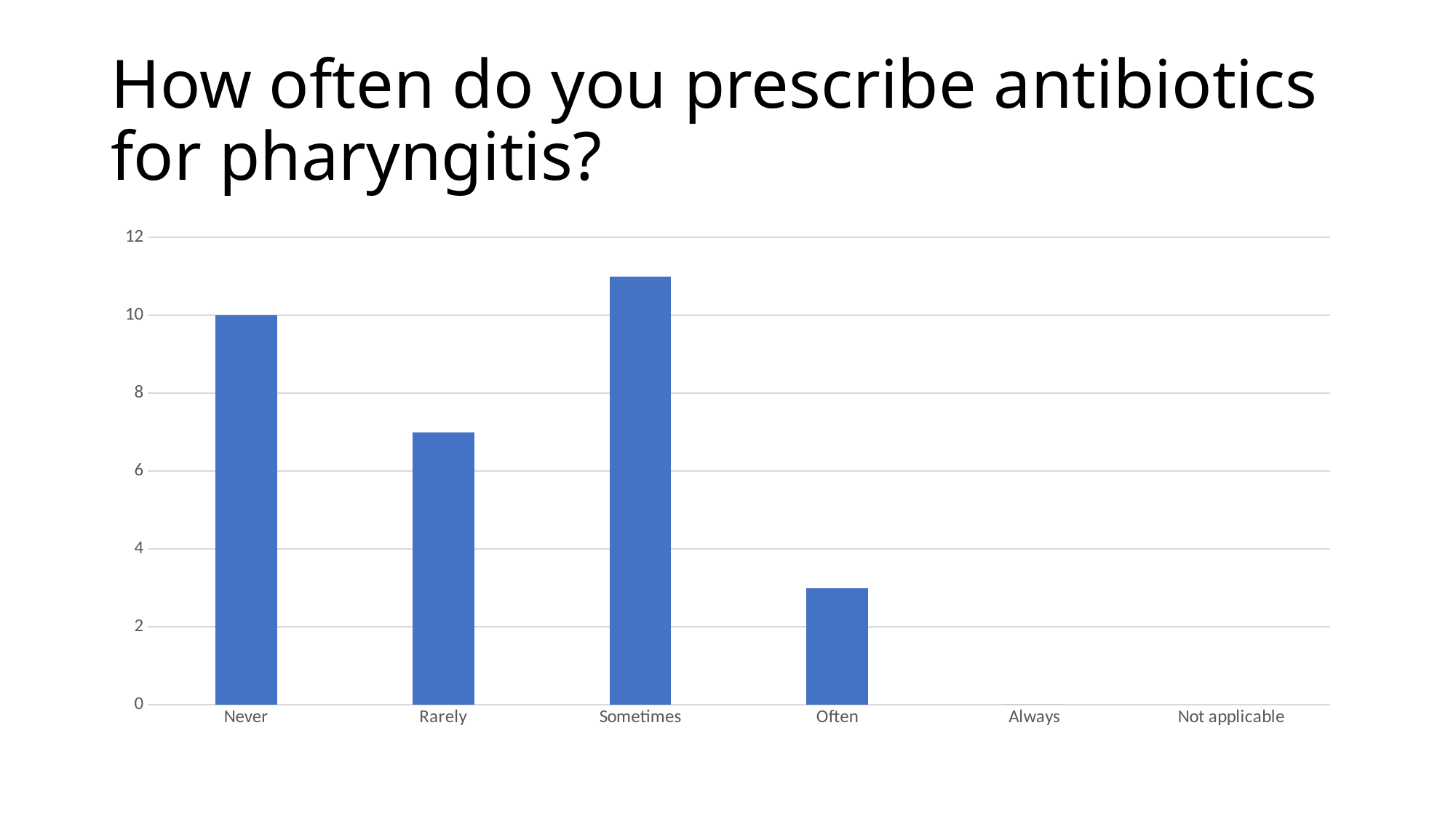

# How often do you prescribe antibiotics for pharyngitis?
### Chart
| Category | Series 1 |
|---|---|
| Never | 10.0 |
| Rarely | 7.0 |
| Sometimes | 11.0 |
| Often | 3.0 |
| Always | 0.0 |
| Not applicable | None |

## Slide 10
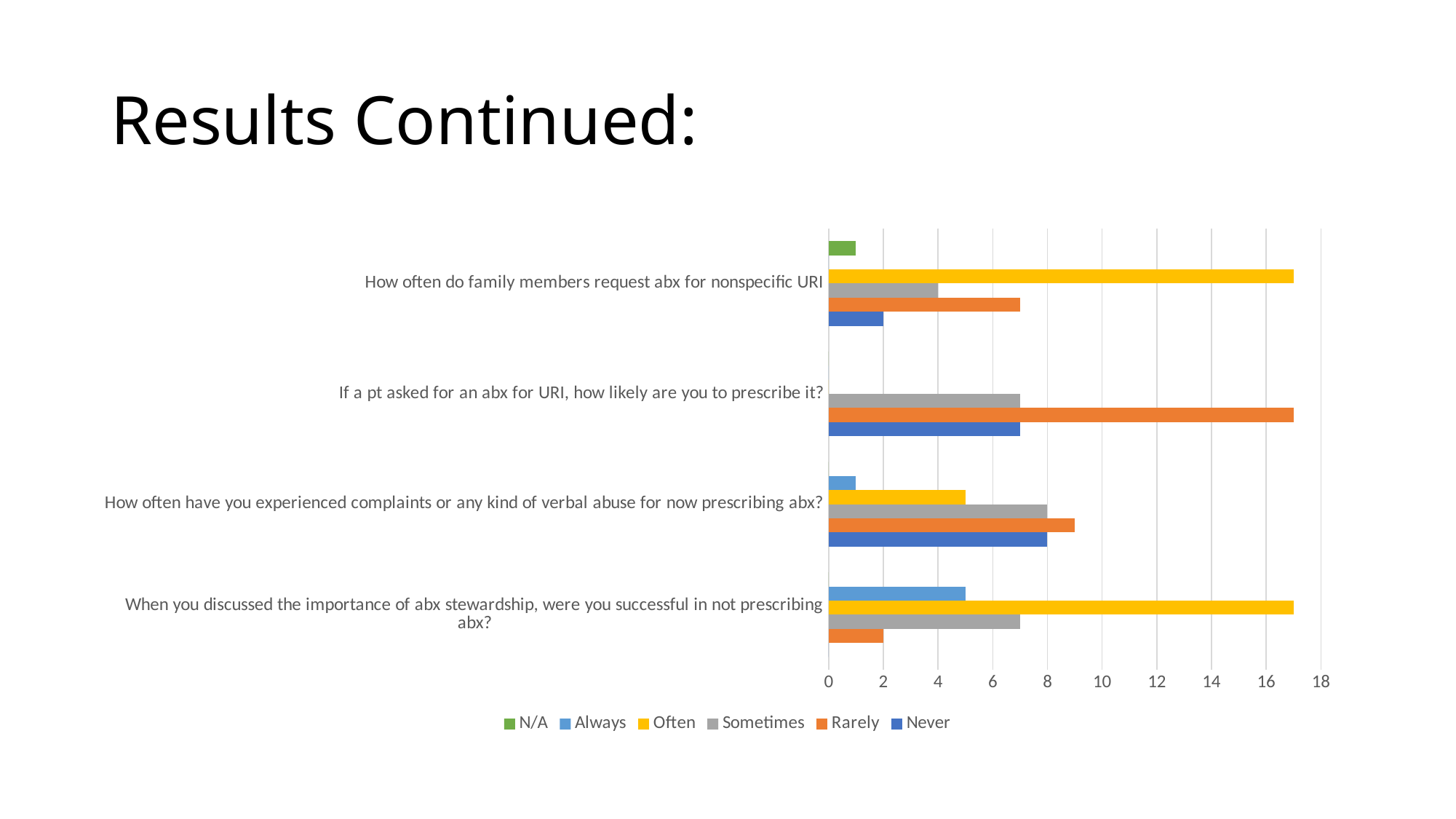

# Results Continued:
### Chart
| Category | Never | Rarely | Sometimes | Often | Always | N/A |
|---|---|---|---|---|---|---|
| When you discussed the importance of abx stewardship, were you successful in not prescribing abx? | 0.0 | 2.0 | 7.0 | 17.0 | 5.0 | 0.0 |
| How often have you experienced complaints or any kind of verbal abuse for now prescribing abx? | 8.0 | 9.0 | 8.0 | 5.0 | 1.0 | 0.0 |
| If a pt asked for an abx for URI, how likely are you to prescribe it? | 7.0 | 17.0 | 7.0 | 0.0 | 0.0 | 0.0 |
| How often do family members request abx for nonspecific URI | 2.0 | 7.0 | 4.0 | 17.0 | 0.0 | 1.0 |

## Slide 11
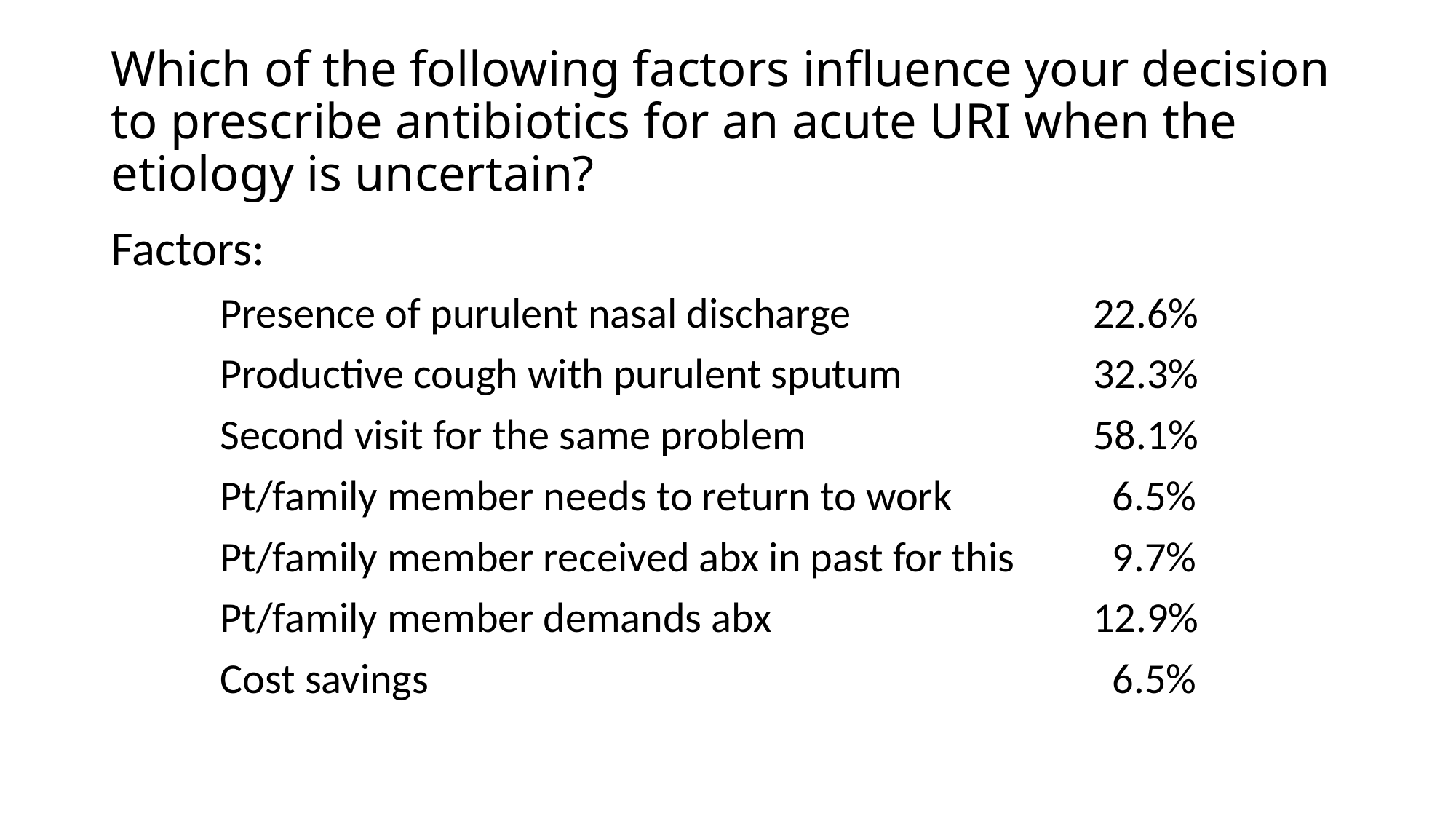

# Which of the following factors influence your decision to prescribe antibiotics for an acute URI when the etiology is uncertain?
Factors:
	Presence of purulent nasal discharge 			22.6%
	Productive cough with purulent sputum		32.3%
 	Second visit for the same problem			58.1%
	Pt/family member needs to return to work		 6.5%
	Pt/family member received abx in past for this	 9.7%
	Pt/family member demands abx			12.9%
	Cost savings							 6.5%

## Slide 12
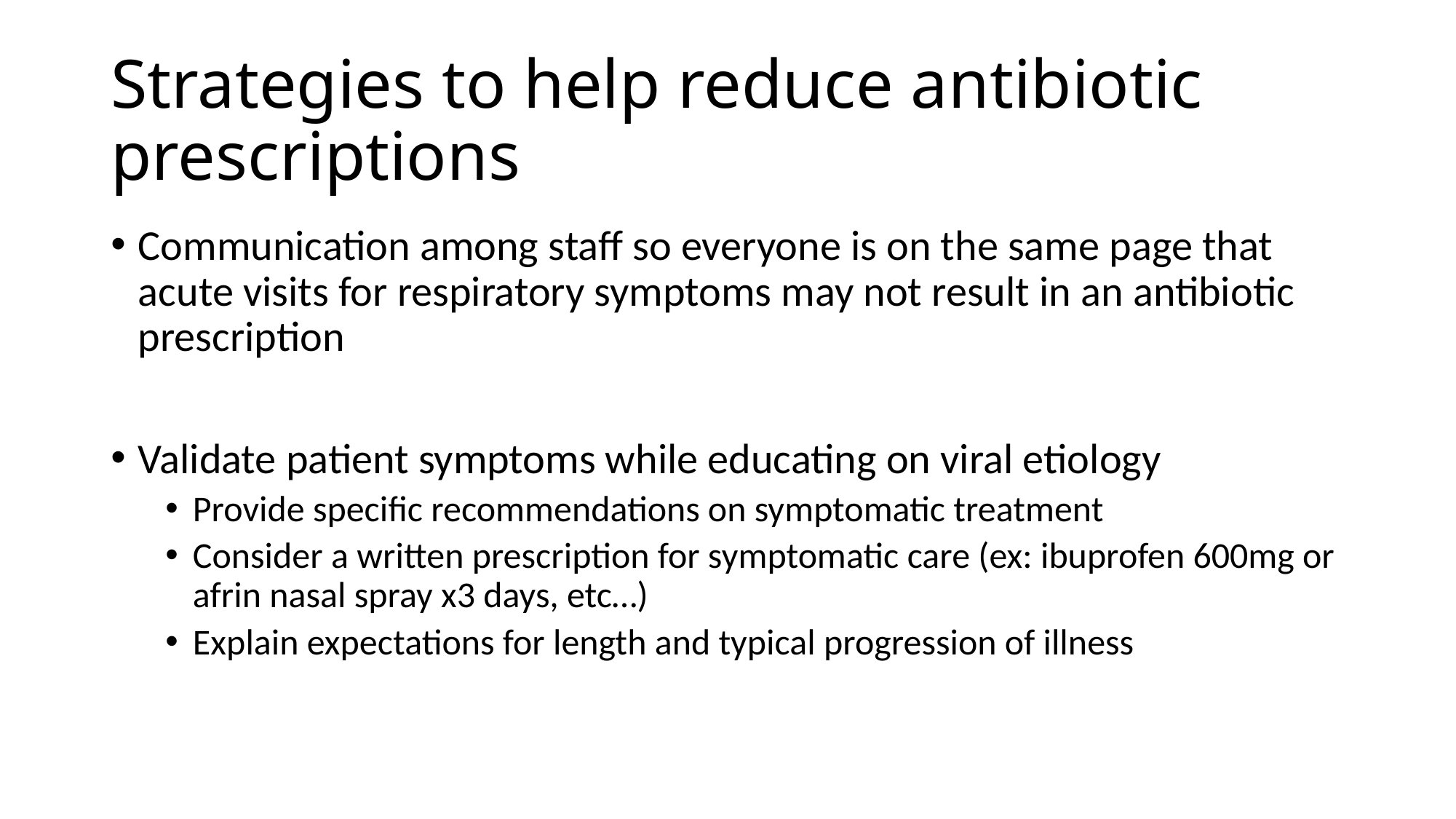

# Strategies to help reduce antibiotic prescriptions
Communication among staff so everyone is on the same page that acute visits for respiratory symptoms may not result in an antibiotic prescription
Validate patient symptoms while educating on viral etiology
Provide specific recommendations on symptomatic treatment
Consider a written prescription for symptomatic care (ex: ibuprofen 600mg or afrin nasal spray x3 days, etc…)
Explain expectations for length and typical progression of illness

## Slide 13
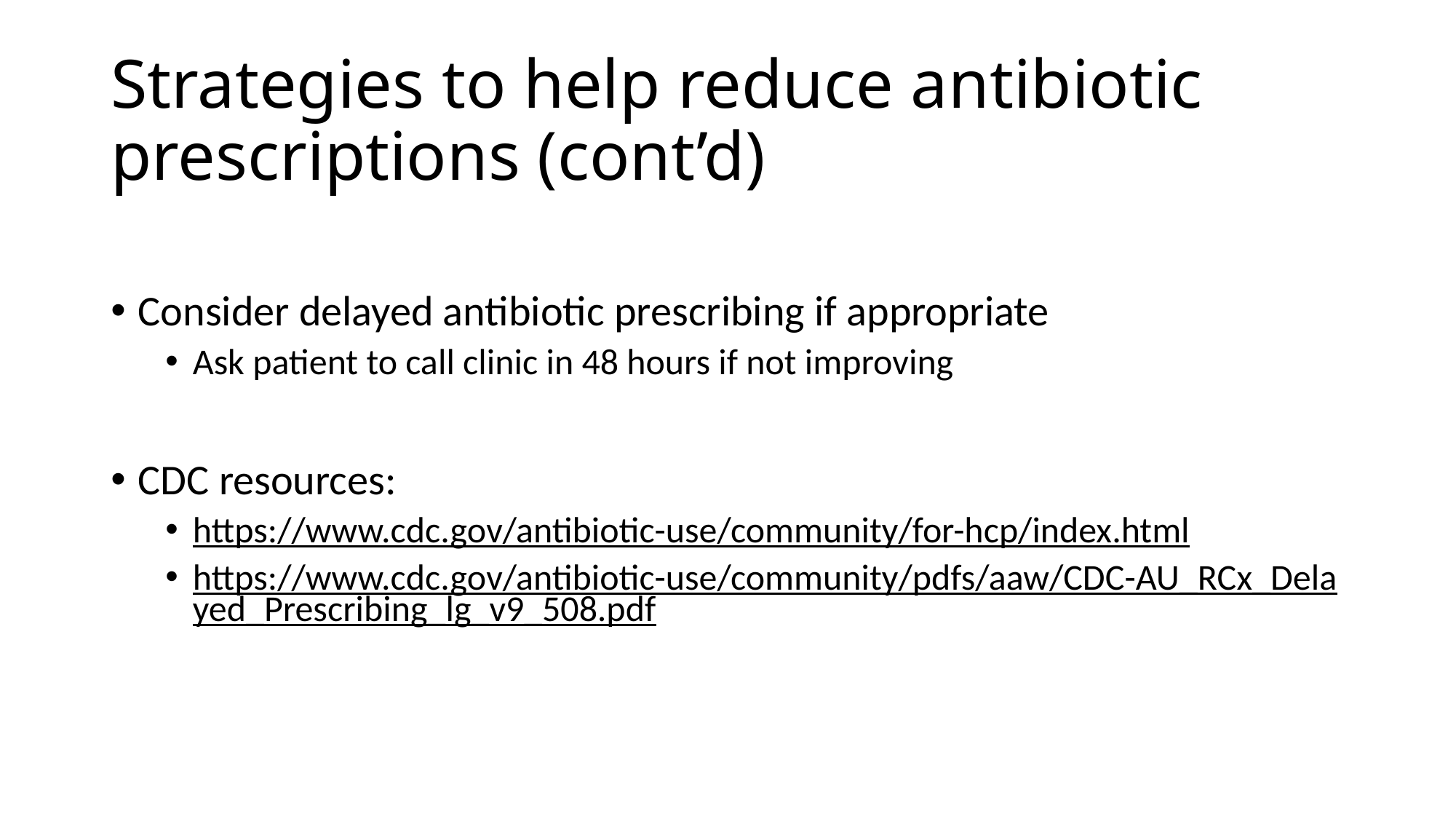

# Strategies to help reduce antibiotic prescriptions (cont’d)
Consider delayed antibiotic prescribing if appropriate
Ask patient to call clinic in 48 hours if not improving
CDC resources:
https://www.cdc.gov/antibiotic-use/community/for-hcp/index.html
https://www.cdc.gov/antibiotic-use/community/pdfs/aaw/CDC-AU_RCx_Delayed_Prescribing_lg_v9_508.pdf

## Slide 14
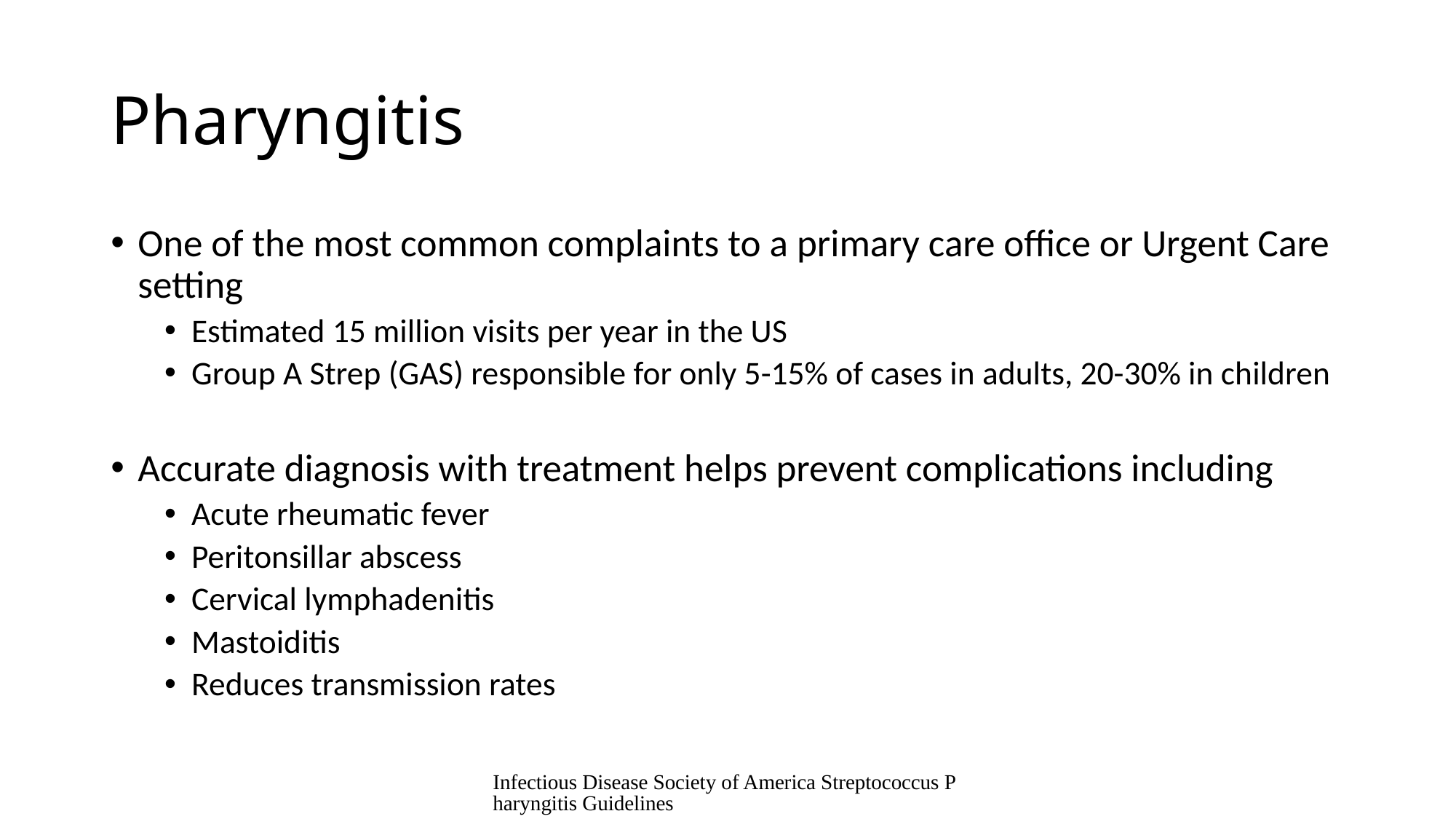

# Pharyngitis
One of the most common complaints to a primary care office or Urgent Care setting
Estimated 15 million visits per year in the US
Group A Strep (GAS) responsible for only 5-15% of cases in adults, 20-30% in children
Accurate diagnosis with treatment helps prevent complications including
Acute rheumatic fever
Peritonsillar abscess
Cervical lymphadenitis
Mastoiditis
Reduces transmission rates
Infectious Disease Society of America Streptococcus Pharyngitis Guidelines

## Slide 15
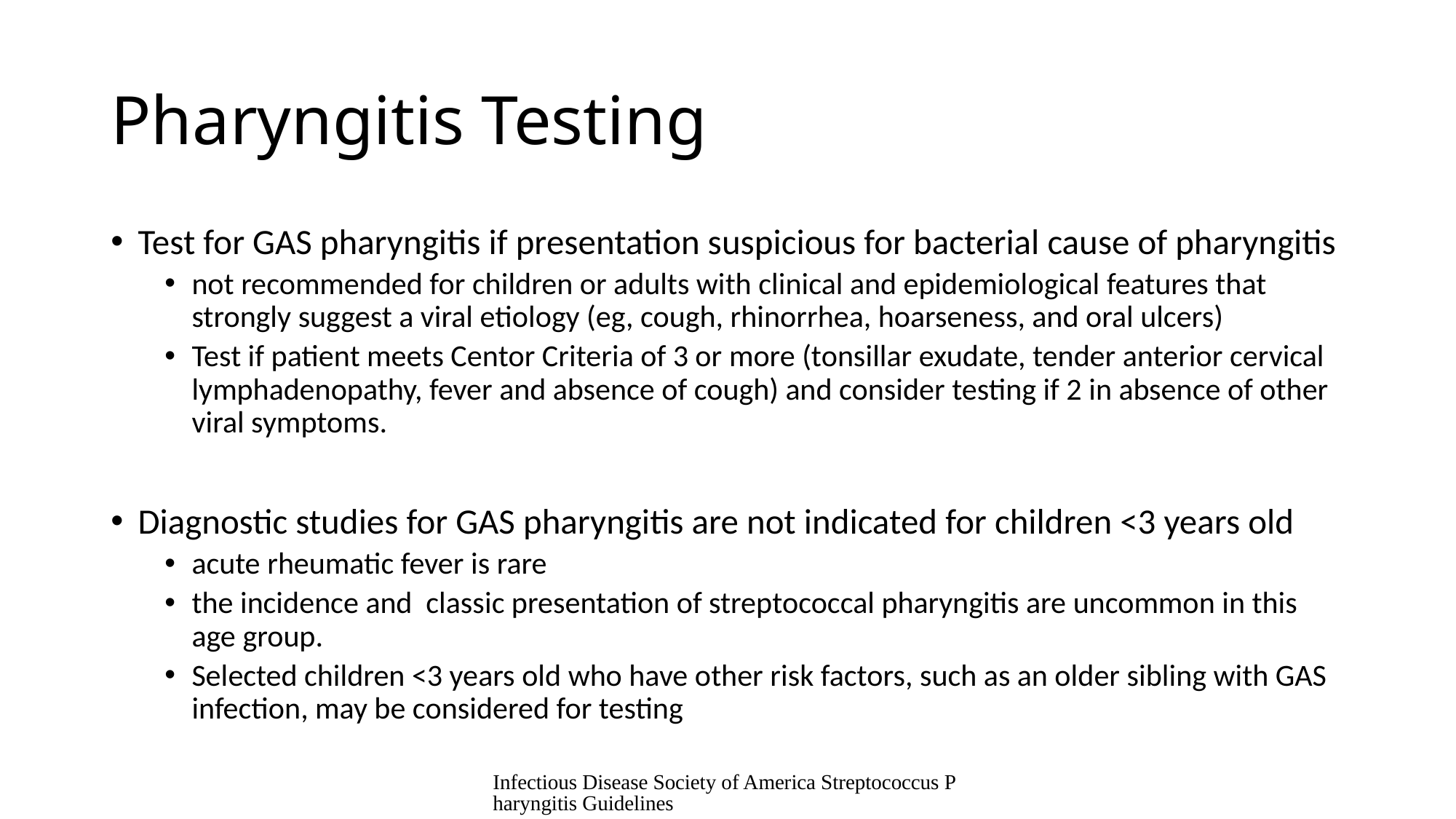

# Pharyngitis Testing
Test for GAS pharyngitis if presentation suspicious for bacterial cause of pharyngitis
not recommended for children or adults with clinical and epidemiological features that strongly suggest a viral etiology (eg, cough, rhinorrhea, hoarseness, and oral ulcers)
Test if patient meets Centor Criteria of 3 or more (tonsillar exudate, tender anterior cervical lymphadenopathy, fever and absence of cough) and consider testing if 2 in absence of other viral symptoms.
Diagnostic studies for GAS pharyngitis are not indicated for children <3 years old
acute rheumatic fever is rare
the incidence and classic presentation of streptococcal pharyngitis are uncommon in this age group.
Selected children <3 years old who have other risk factors, such as an older sibling with GAS infection, may be considered for testing
Infectious Disease Society of America Streptococcus Pharyngitis Guidelines

## Slide 16
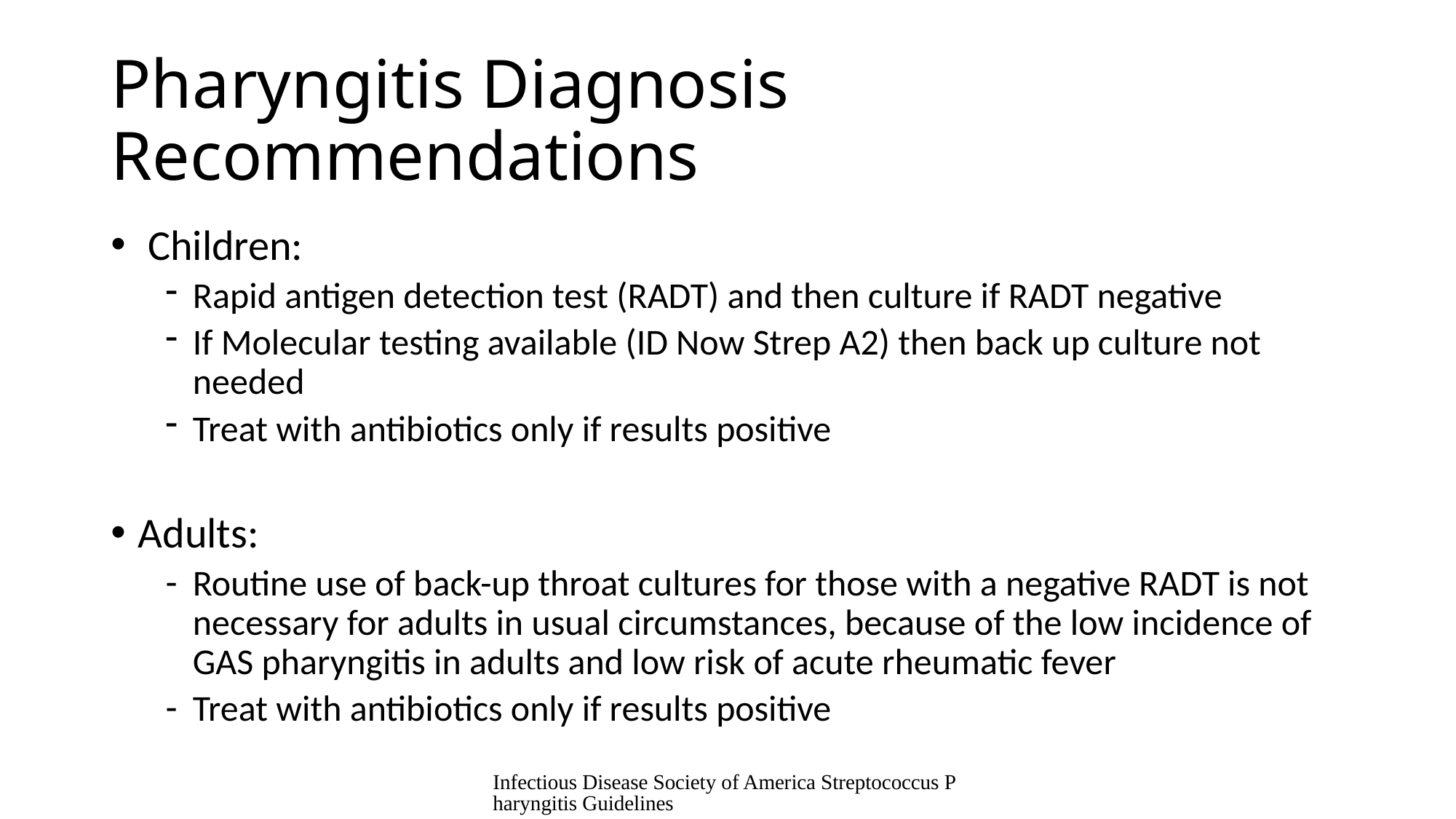

# Pharyngitis Diagnosis Recommendations
 Children:
Rapid antigen detection test (RADT) and then culture if RADT negative
If Molecular testing available (ID Now Strep A2) then back up culture not needed
Treat with antibiotics only if results positive
Adults:
Routine use of back-up throat cultures for those with a negative RADT is not necessary for adults in usual circumstances, because of the low incidence of GAS pharyngitis in adults and low risk of acute rheumatic fever
Treat with antibiotics only if results positive
Infectious Disease Society of America Streptococcus Pharyngitis Guidelines

## Slide 17
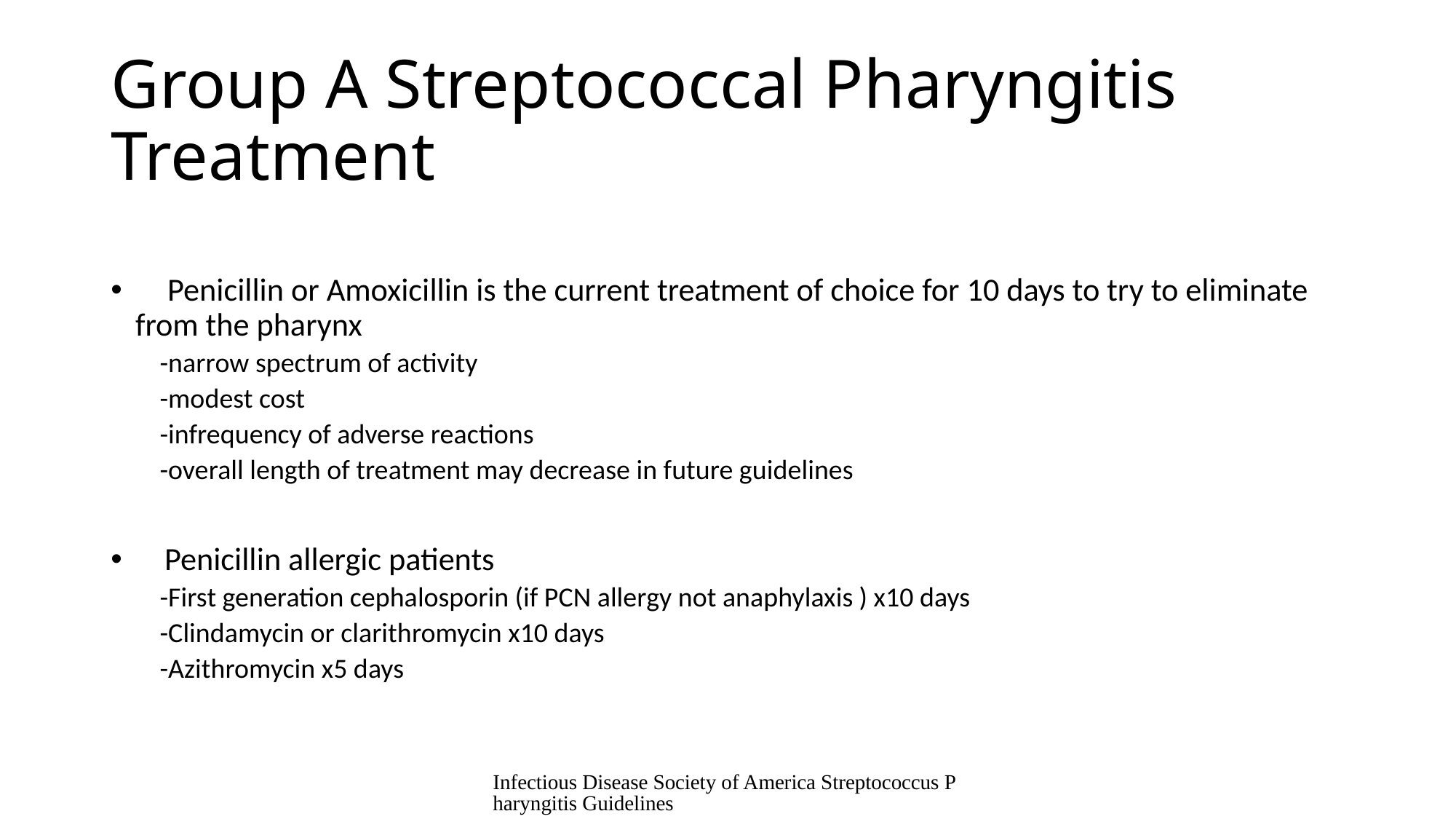

# Group A Streptococcal Pharyngitis Treatment
 Penicillin or Amoxicillin is the current treatment of choice for 10 days to try to eliminate from the pharynx
-narrow spectrum of activity
-modest cost
-infrequency of adverse reactions
-overall length of treatment may decrease in future guidelines
 Penicillin allergic patients
-First generation cephalosporin (if PCN allergy not anaphylaxis ) x10 days
-Clindamycin or clarithromycin x10 days
-Azithromycin x5 days
Infectious Disease Society of America Streptococcus Pharyngitis Guidelines

## Slide 18
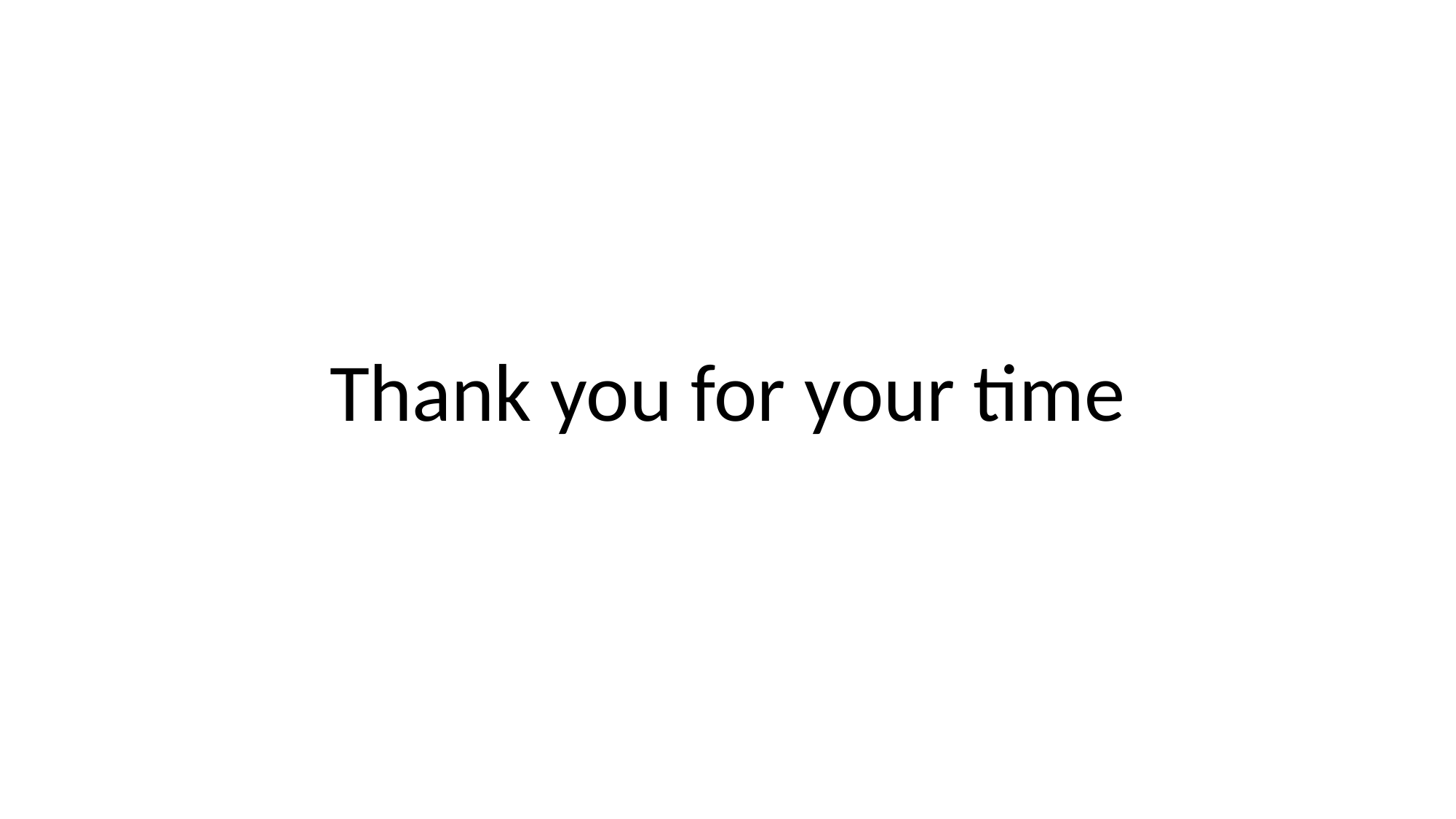

Thank you for your time

## Slide 19
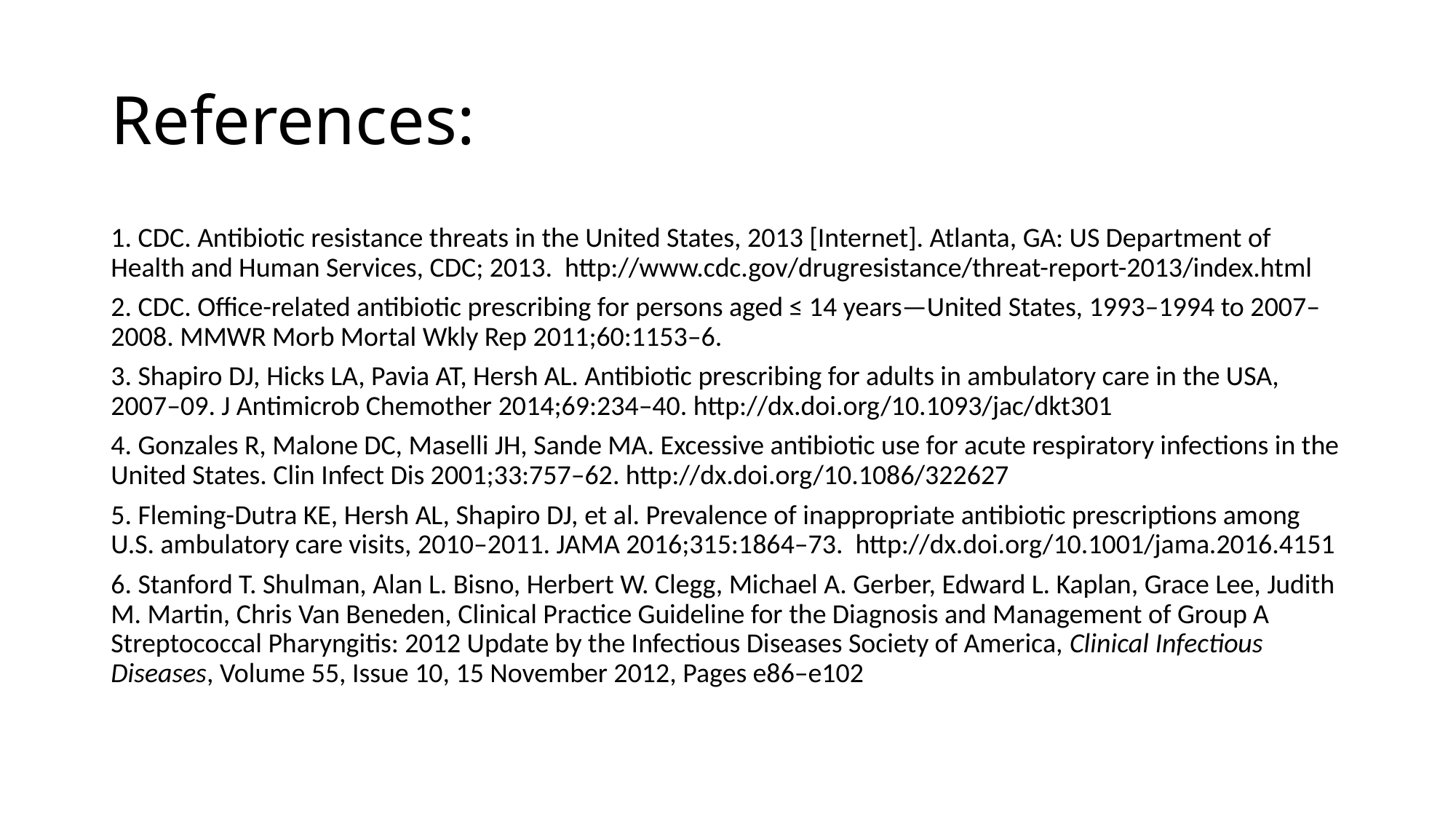

# References:
1. CDC. Antibiotic resistance threats in the United States, 2013 [Internet]. Atlanta, GA: US Department of Health and Human Services, CDC; 2013. http://www.cdc.gov/drugresistance/threat-report-2013/index.html
2. CDC. Office-related antibiotic prescribing for persons aged ≤ 14 years—United States, 1993–1994 to 2007–2008. MMWR Morb Mortal Wkly Rep 2011;60:1153–6.
3. Shapiro DJ, Hicks LA, Pavia AT, Hersh AL. Antibiotic prescribing for adults in ambulatory care in the USA, 2007–09. J Antimicrob Chemother 2014;69:234–40. http://dx.doi.org/10.1093/jac/dkt301
4. Gonzales R, Malone DC, Maselli JH, Sande MA. Excessive antibiotic use for acute respiratory infections in the United States. Clin Infect Dis 2001;33:757–62. http://dx.doi.org/10.1086/322627
5. Fleming-Dutra KE, Hersh AL, Shapiro DJ, et al. Prevalence of inappropriate antibiotic prescriptions among U.S. ambulatory care visits, 2010–2011. JAMA 2016;315:1864–73. http://dx.doi.org/10.1001/jama.2016.4151
6. Stanford T. Shulman, Alan L. Bisno, Herbert W. Clegg, Michael A. Gerber, Edward L. Kaplan, Grace Lee, Judith M. Martin, Chris Van Beneden, Clinical Practice Guideline for the Diagnosis and Management of Group A Streptococcal Pharyngitis: 2012 Update by the Infectious Diseases Society of America, Clinical Infectious Diseases, Volume 55, Issue 10, 15 November 2012, Pages e86–e102
