# Supplementary material for: Introducing antimicrobial stewardship to the outpatient clinics of a suburban academic health system
Source: Antimicrob Steward Healthc Epidemiol. Author manuscript; Available in PMC 2022 Jul 26. (PMC9319114; doi:10.1017/ash.2021.228)
Supplement: Supplementary file 1 [file NIHMS1817803-supplement-Supplemental_Figure_1.pdf]

Q01 - Are you:							
Physician							
Advanced Practice Nurse							
Physician's Assistant							
Others							
Skipped							

Q02 - Which of the following best describes your primary practice setting?							
Immediate/Urgent Care Clinic							
Primary Care Clinic for adults only							
Primary Care Clinic for both adults and pediatrics							
Primary Care Clinic for pediatrics only							
Multidisciplinary Clinic							
Others							
Skip							
Comments							

Q03 - What is your specialty?							
Family Medicine							
Internal Medicine							
Emergency Medicine							
Pediatrics							
Medicine-Pediatrics							
Other							
Skip							

Q04 - Knowledge and perception of antimicrobial stewardship and resistance:

	Strongly Agree	Agree	Unsure/ Neither Agree nor Disagree	Disagree	Strongly Disagree	Total	Weighted Average
Q04a - Antibiotics are overprescribed nationally.							
Q04b - Antibiotics are overprescribed in my primary practice.							
Q04c - I prescribe more antibiotics than I should.							
Q04d - I am concerned about antibiotic resistance in the community when I prescribe antibiotics.							
Q04e - My patients think I should prescribe antibiotics for cough, cold, or flu symptoms.							
Q04f - I can reduce my own antibiotic prescribing without any decrease in patient satisfaction.							
Q04g - Prescribing antibiotics for acute bronchitis is an accepted standard of care in my facility/practice.							
Q04h - I prescribe antibiotics based on telephonic assessment of the patient.							
Q04i - I am concerned about complications when I prescribe antibiotics including *C. difficile* infection.							

Q04j - Use of rapid diagnostic testing (rapid strep, influenza, respiratory PCR) can impact patient care.								

Comments

Q05 - Provider prescription of antibiotics:

	Never	Rarely	Some-times	Often	Always	N/A	Total	Weighted Average
Q05a - If a patient/family member asked you to prescribe antibiotics for a non-specific upper respiratory tract infection (URI), how often are you comfortable explaining why antibiotics do not work for certain conditions?								
Q05b - If a patient/family member insisted that you prescribe antibiotics for a non-specific URI how likely is it that you would prescribe antibiotics?								
Q05c - How often do patients/family members ask you to prescribe antibiotics for a non-specific URI?								
Q05d - How often do you have conversations (i.e., discussion) with your patients/family members about prescribing antibiotics for a non-specific URI?								
Q05e - How often have you experienced complaints threats or any kind of verbal abuse from a patient or family member for not prescribing an antibiotic?								
Q05f - How often do you prescribe antibiotics for non-specific upper respiratory tract symptoms?								
Q05g - How often do you prescribe antibiotics for acute bronchitis?								
Q05h - How often do you prescribe antibiotics B44for pharyngitis?								
Q05i - When you discussed the importance of antibiotic stewardship, were you successful in not prescribing antibiotics?								
Q05j - How often do you obtain rapid influenza and respiratory PCR?								
Q05k - Do you prescribe/continue antibiotics when the rapid influenza/respiratory PCR shows a viral etiology?								

Comments

strep + will treat

Q06 - Which of the following factor(s) influence your decision to prescribe an antibiotic for an acute upper respiratory infection (URI) when the etiology (viral vs. bacterial) is uncertain? Check all that apply.

Presence of purulent nasal discharge
Productive cough with purulent sputum
Second visit for the same problem
Patient/family member needs to return to work

Patient/family member states that he/she received an antibiotic for similar symptoms in the past

Patient/family member demands or expects to receive antibiotics

Cost savings for the patient/family member

Please list any other factors that influence your decision to prescribe antibiotics.

Q07 - Delayed Prescribing Strategies. Delayed prescribing refers to a strategy in which a patient is asked to wait (usually 24-48 hours) after a clinical visit to determine if an antibiotic is needed.

	Never	Rarely	Some-times	Often	Always	N/A	Total	Weighted Average
In the past year, how often have you used delayed prescribing for patients with an acute respiratory infection when the etiology (viral vs. bacterial) was uncertain?								

Comments

Q08 - In the past year, how have you used delayed prescribing for patients with an acute respiratory infection when the etiology (viral vs. bacterial) was uncertain? Select all that apply.

Include a "Do not fill until" date on the prescription.

Re-Contact patient to provide a prescription.

Instruct patient to call if symptoms persist.

Instruct patient to return to clinic if symptoms persist.

I did not use delayed prescribing in the past year.

Other (please specify)

Q09 - Patient Education. Which of the following patient education resources on appropriate antibiotic prescribing would you use if provided? Select all that apply.

Patient education handouts (e.g., fact sheets, brochures) downloaded/printed from the internet.

Patient education handouts (e.g., fact sheets, brochures) delivered to your facility in hard copy.

Patient education handouts (e.g., fact sheets, brochures) developed by your facility.

Waiting room videos.

Tear-off sheets or prescription pads for symptomatic relief for viral illness (i.e., home or over the counter remedies).

EPIC embedded patient instructions.

Other (please specify)

Q10 - Provider Education. What topics related to antibiotic use would you like to learn more about? Please select all that apply.

Epidemiology of antibiotic resistance

Review of best practices for improving antibiotic prescribing in outpatient settings

Rapid diagnostics
Treatment guidelines for acute respiratory tract infections
Strategies to improve patient satisfaction when antibiotics are unnecessary
Measuring impact of antibiotic stewardship
Data comparing antibiotic prescribing use of me to my peers
Benchmarking antibiotic stewardship outcomes
Electronic data capture systems
Pertinent national and state policies
Other (please specify)
