# Supplementary material for: Introducing antimicrobial stewardship to the outpatient clinics of a suburban academic health system
Source: Antimicrob Steward Healthc Epidemiol. Author manuscript; Available in PMC 2022 Jul 26. (PMC9319114; doi:10.1017/ash.2021.228)
Supplement: Supplementary file 2 [file NIHMS1817803-supplement-Supplemental_Figure_2A.pdf]

From: [redacted]
Sent: Wednesday, May 26, 2021 10:53:56 AM
To: [redacted]
Subject: Primary Care Quality-PR

On the overall **Primary Care Quality** report through the end of April, some notables:

- Ongoing strong improvement in:
  - Antibiotic stewardship (our best month yet)
  - Pediatric measures (excluding our 0-15 months subset)
  - Prevention & screening in adults
- Improvement in:
  - <u>Hypertension control at 68%.</u> Although not yet back to pre-pandemic levels as a service line, we have seen improvement in your HTN control for the last 2 months. Our goal is 80%.

Thank you for all your hard work and attention to quality in light of the challenges faced across ambulatory in the last 12 months.

Please let me know if you have any questions/concerns.

Thanks
[redacted]

[redacted]

Overall_April 2021.xlsx

[redacted]-April.xlsx
