# Supplementary material for: Introducing antimicrobial stewardship to the outpatient clinics of a suburban academic health system
Source: Antimicrob Steward Healthc Epidemiol. Author manuscript; Available in PMC 2022 Jul 26. (PMC9319114; doi:10.1017/ash.2021.228)
Supplement: Supplementary file 3 [file NIHMS1817803-supplement-Supplemental_Figure_2B.pdf]

From: [redacted]
Sent: Monday, June 14, 2021 9:32:08 AM
To: [redacted]
Subject: May Quality - [redacted]

Attached are three reports representing quality performance for Primary Care overall as well as for [redacted] specifically with notes for each:

- Overall **Primary Care Quality**:
  - Antibiotic Stewardship: great job with improvements over the last year; keep it going!
  - Pediatric: 4 of the 5 measures performance remained the same or higher
  - Prevention & Screening: 6 of the 7 measures remained the same or higher
  - Chronic diseases:
    - Diabetes saw *improvemen*t in blood pressure control and A1C with opportunities to improve capturing eye exams
    - Hypertension control saw incremental *improvements;* incredible efforts by all!

- [redacted] **Quality** with details by provider
  - Providers that are high performers may have best practices to share

- **TargetBP biweekly**
  - The challenge remains to facilitate timely follow-up appointments (either in-person or through telehealth) to gather BP readings as a response to medication intensification

[redacted]

Overall_May 2021.xlsx

[redacted].xlsx

[redacted]...21.xlsx
