# Supplementary material for: Introducing antimicrobial stewardship to the outpatient clinics of a suburban academic health system
Source: Antimicrob Steward Healthc Epidemiol. Author manuscript; Available in PMC 2022 Jul 26. (PMC9319114; doi:10.1017/ash.2021.228)
Supplement: Supplementary file 4 [file NIHMS1817803-supplement-Supplemental_Figure_3A.pdf]

Loyola Physician Partners - LUMC Employed Primary Care
Quality Measure Results

	Target*	2020 May	2020 June	2020 July	2020 Aug	2020 Sep	2020 Oct	2020 Nov	2020 Dec	2021 Jan	2021 Feb	2021 Mar	2021 Apr	2021 May	Trend
**Pediatric Wellness and Prevention[1]**															
Well Visits in the First 15 months	**86.3%**	66.7%	68.0%	67.1%	66.8%	65.8%	64.7%	64.5%	63.4%	63.2%	64.3%	63.9%	63.7%	63.3%	
Well Visits Ages 3 to 6	**83.0%**	78.9%	78.3%	79.8%	78.9%	79.5%	81.0%	81.0%	80.7%	80.1%	80.7%	81.3%	84.2%	84.1%	
Well Visits Adolescent		67.5%	65.6%	64.8%	64.0%	64.5%	65.8%	66.5%	66.9%	66.9%	68.3%	69.6%	72.3%	73.3%	
Depression Screen for Adolescents	**90.0%**	72.8%	71.8%	71.5%	71.1%	71.1%	71.5%	72.2%	71.6%	71.0%	71.5%	73.5%	77.2%	78.6%	
BMI Screening for Pediatrics	**90.0%**	79.4%	79.1%	80.1%	80.5%	81.2%	82.0%	81.9%	81.0%	80.3%	80.6%	81.0%	83.8%	84.5%	
**Prevention and Screening - Women's Health**															
Breast Cancer Screening	**77.3%**	77.8%	77.5%	77.1%	77.1%	77.2%	77.1%	76.9%	76.8%	76.4%	76.3%	76.2%	75.8%	76.1%	
Cervical Cancer Screening	**78.0%**	70.6%	70.5%	70.6%	70.3%	70.6%	70.9%	71.1%	71.3%	71.0%	70.9%	71.0%	71.1%	71.1%	
**Prevention and Screening - Adults**															
BMI Screening	**90.0%**	79.1%	78.8%	78.4%	78.5%	78.0%	78.1%	77.6%	76.7%	75.4%	75.1%	75.9%	78.8%	80.3%	
Colorectal Cancer Screening	**80.0%**	73.4%	73.3%	73.3%	73.5%	73.6%	73.8%	73.9%	74.0%	73.9%	73.8%	73.6%	73.6%	73.7%	
Falls Risk Screening	**90.0%**	81.2%	81.1%	81.1%	81.1%	81.6%	81.6%	81.8%	81.5%	80.9%	81.0%	83.2%	85.6%	86.5%	
Tobacco Screening	**92.3%**	95.8%	95.9%	96.0%	96.1%	96.2%	96.2%	96.2%	96.3%	96.4%	96.5%	96.6%	96.6%	96.6%	
Depression Screening for Adults	**90.0%**	74.0%	74.0%	74.1%	74.5%	74.8%	74.9%	75.5%	75.5%	75.5%	75.7%	77.4%	79.9%	81.3%	
**Chronic Diseases - Diabetes[2]**															
HbA1c <8 (Higher is better)	**79.0%**	61.5%	61.5%	61.5%	61.6%	61.6%	61.6%	62.0%	62.4%	62.0%	62.1%	62.5%	63.6%	64.1%	
Annual Dilated Retinal Exam	**63.5%**	53.7%	53.1%	52.7%	52.2%	51.8%	51.2%	50.7%	50.5%	49.5%	49.2%	48.5%	48.0%	47.8%	
Blood Pressure < 140/90	**72.5%**	73.6%	73.5%	74.1%	74.0%	73.3%	72.3%	71.5%	71.4%	71.1%	70.6%	70.5%	71.6%	72.6%	
**Chronic Diseases - Hypertension[3]**															
Blood Pressure < 140/90	**80.0%**	72.3%	72.0%	71.5%	71.1%	70.5%	69.5%	68.6%	67.4%	66.3%	65.8%	66.3%	68.0%	68.3%	
**Antibiotic Stewardship**															
Appropriate Antibiotic Tx for Children with Pharyngitis	**85.5%**	81.7%	85.3%	85.4%	82.3%	82.7%	83.8%	83.9%	86.0%	85.8%	85.5%	89.4%	89.6%	90.8%	
Appropriate Treatment for Children with URI	**89.8%**	89.5%	89.3%	89.4%	89.5%	89.9%	92.1%	91.7%	91.9%	91.8%	91.3%	93.3%	93.7%	94.7%	
Avoidance of Antibiotic Treatment in Adults with Bronchitis	**24.5%**	90.2%	90.5%	91.4%	91.5%	92.3%	93.0%	93.4%	94.8%	95.9%	96.1%	97.5%	97.0%	97.0%	

Shaded cell means revised and validated metric
