# Supplementary material for: Introducing antimicrobial stewardship to the outpatient clinics of a suburban academic health system
Source: Antimicrob Steward Healthc Epidemiol. Author manuscript; Available in PMC 2022 Jul 26. (PMC9319114; doi:10.1017/ash.2021.228)
Supplement: Supplementary file 5 [file NIHMS1817803-supplement-Supplemental_Figure_3B.pdf]

Loyola Physician Partners - Employed
Quality Measure Results - May 2021

	Target *	[redacted]	[redacted]	[redacted]	[redacted]	[redacted]	[redacted]	[redacted]	[redacted]
**Pediatric Wellness and Prevention[1]**									
Well Visits in the First 15 months	86.3%	50.0% (14/28)	69.2% (9/13)	66.7% (2/3)	70.6% (12/17)	75.0% (21/28)		67.9% (19/28)	90.5% (19/21)
Well Visits Ages 3 to 6	83.0%	86.8% (112/129)	78.6% (77/98)	76.5% (13/17)	83.8% (57/68)	84.4% (151/179)	100.0% (2/2)	74.8% (86/115)	86.6% (84/97)
Well Visits Adolescent		81.5% (110/135)	76.0% (130/171)	77.2% (44/57)	72.5% (166/229)	76.5% (228/298)	75.8% (25/33)	71.1% (192/270)	65.0% (80/123)
Depression Screen for Adolescents	90.0%	83.3% (55/66)	67.1% (51/76)	61.5% (24/39)	64.7% (66/102)	61.3% (84/137)	63.6% (7/11)	65.1% (95/146)	83.0% (44/53)
BMI Screening for Pediatrics	90.0%	89.9% (726/808)	53.1% (373/702)	49.1% (105/214)	58.8% (438/745)	63.8% (877/1375)	69.0% (40/58)	55.2% (465/843)	75.1% (368/490)
**Prevention and Screening - Women's Health**									
Breast Cancer Screening	77.3%	77.3% (245/317)	74.7% (251/336)	92.2% (71/77)	66.5% (133/200)	77.7% (327/421)	77.3% (116/150)	56.6% (69/122)	72.7% (250/344)
Cervical Cancer Screening	78.0%	65.7% (241/367)	75.7% (418/552)	62.0% (57/92)	57.1% (124/217)	64.6% (411/636)	56.0% (51/91)	62.9% (124/197)	67.4% (358/531)
**Prevention and Screening - Adults**									
BMI Screening	90.0%	88.0% (889/1010)	78.0% (864/1107)	82.7% (349/422)	76.0% (865/1138)	78.2% (864/1105)	88.0% (559/635)	79.5% (784/986)	79.5% (820/1031)
Colorectal Cancer Screening	80.0%	73.7% (404/548)	68.5% (357/521)	86.8% (217/250)	70.9% (436/615)	68.3% (362/530)	81.0% (319/394)	62.9% (283/450)	70.3% (344/489)
Falls Risk Screening	90.0%	86.1% (304/353)	88.7% (205/231)	89.8% (141/157)	83.8% (320/382)	86.7% (208/240)	93.4% (311/333)	83.5% (187/224)	87.1% (217/249)
Tobacco Screening	92.3%	97.6% (969/993)	97.0% (1039/1071)	97.3% (400/411)	97.7% (1058/1083)	97.5% (1042/1069)	99.0% (611/617)	96.5% (907/940)	97.1% (984/1013)
Depression Screening for Adults	90.0%	87.0% (724/832)	78.2% (654/836)	80.5% (309/384)	77.2% (753/976)	78.1% (676/866)	88.8% (477/537)	77.1% (653/847)	81.1% (647/798)
**Chronic Diseases - Diabetes[2]**									
HbA1c <8 (Higher is better)	79.0%	73.1% (95/130)	66.4% (101/152)	75.4% (52/69)	60.3% (114/189)	60.2% (59/98)	64.3% (72/112)	60.7% (71/117)	73.5% (100/136)
Annual Dilated Retinal Exam	63.5%	55.4% (72/130)	34.2% (52/152)	44.9% (31/69)	36.0% (68/189)	34.7% (34/98)	60.7% (68/112)	30.8% (36/117)	50.7% (69/136)
Blood Pressure < 140/90	72.5%	72.3% (94/130)	70.4% (107/152)	73.9% (51/69)	68.8% (130/189)	83.7% (82/98)	71.4% (80/112)	70.1% (82/117)	74.3% (101/136)
**Chronic Diseases - Hypertension[3]**									
Blood Pressure < 140/90	80.0%	74.3% (349/470)	64.5% (262/406)	78.3% (188/240)	60.0% (306/510)	76.4% (249/326)	67.2% (242/360)	58.3% (200/343)	65.7% (216/329)
**Antibiotic Stewardship**									
Appropriate Antibiotic Tx for Children with Pharyngitis	85.5%		100.0% (2/2)	66.7% (2/3)	100.0% (6/6)	71.4% (5/7)	100.0% (2/2)	100.0% (2/2)	80.0% (4/5)
Appropriate Treatment for Children with URI	89.8%				100.0% (1/1)		100.0% (1/1)		100.0% (1/1)
Avoidance of Antibiotic Treatment in Adults with Bronchitis	24.5%	100.0% (4/4)	100.0% (9/9)	100.0% (1/1)	100.0% (3/3)	100.0% (11/11)	100.0% (1/1)	100.0% (3/3)	87.5% (7/8)

[1] *Pediatric Wellness and Prevention measures not displayed for internal medicine physicians.*

[2] [3] *Chronic Disease (Diabetes and Hypertension) measure data not displayed for physicians with fewer than 25*
